# Ebola Zaire Virus Blocks Type I Interferon Production by Exploiting the Host SUMO Modification Machinery

**DOI:** 10.1371/journal.ppat.1000493

**Published:** 2009-06-26

**Authors:** Tsung-Hsien Chang, Toru Kubota, Mayumi Matsuoka, Steven Jones, Steven B. Bradfute, Mike Bray, Keiko Ozato

**Affiliations:** 1 Program in Genomics of Differentiation, Eunice Kennedy Shriver National Institute of Child Health and Human Development, National Institutes of Health, Bethesda, Maryland, United States of America; 2 Population and Public Health Branch, National Microbiology Laboratory, Canadian Science Centre for Human and Animal Health, Winnipeg, Manitoba, Canada; 3 United States Army Medical Institute of Infectious Diseases, Fort Detrick, Maryland, United States of America; 4 Integrated Research Facility, National Institute of Allergy and Infectious Diseases, National Institutes of Health, Fort Detrick, Maryland, United States of America; Mount Sinai School of Medicine, United States of America

## Abstract

Ebola Zaire virus is highly pathogenic for humans, with case fatality rates approaching 90% in large outbreaks in Africa. The virus replicates in macrophages and dendritic cells (DCs), suppressing production of type I interferons (IFNs) while inducing the release of large quantities of proinflammatory cytokines. Although the viral VP35 protein has been shown to inhibit IFN responses, the mechanism by which it blocks IFN production has not been fully elucidated. We expressed VP35 from a mouse-adapted variant of Ebola Zaire virus in murine DCs by retroviral gene transfer, and tested for IFN transcription upon Newcastle Disease virus (NDV) infection and toll-like receptor signaling. We found that VP35 inhibited IFN transcription in DCs following these stimuli by disabling the activity of IRF7, a transcription factor required for IFN transcription. By yeast two-hybrid screens and coimmunoprecipitation assays, we found that VP35 interacted with IRF7, Ubc9 and PIAS1. The latter two are the host SUMO E2 enzyme and E3 ligase, respectively. VP35, while not itself a SUMO ligase, increased PIAS1-mediated SUMOylation of IRF7, and repressed *Ifn* transcription. In contrast, VP35 did not interfere with the activation of NF-κB, which is required for induction of many proinflammatory cytokines. Our findings indicate that Ebola Zaire virus exploits the cellular SUMOylation machinery for its advantage and help to explain how the virus overcomes host innate defenses, causing rapidly overwhelming infection to produce a syndrome resembling fulminant septic shock.

## Author Summary

Ebola Zaire virus causes severe hemorrhagic fever in humans that is fatal in almost 90% of cases. The rapid spread of the virus to macrophages and dendritic cells results in the release of high levels of inflammatory cytokines, causing shock and bleeding. The ability of Ebola virus to overwhelm host defenses is believed to result from its suppression of the type I interferon (IFN) response. The Ebola viral protein VP35 is known to block IFN responses, but the precise mechanisms have not been identified. We expressed VP35 in mouse dendritic cells and found that the cells failed to develop a normal IFN response when infected with Newcastle Disease virus. By a yeast two-hybrid system and other biochemical experiments, we showed that the blockade resulted from the conjugation of a Small Ubiquitin-like Modifier (SUMO) protein to IRF-7, the principal cellular factor required for IFN gene expression. However, the cells were still able to activate NF-κB, a transcription factor responsible for the release of proinflammatory cytokines. Our findings provide a first example where a virus hijacks the host SUMO system to undermine innate immunity, and help to explain how Ebola virus spreads rapidly in lymphoid tissues to cause a lethal inflammatory syndrome.

## Introduction

Ebola Zaire virus (EBOV) causes severe hemorrhagic fever in humans, with case fatality rates as high as 90% in large outbreaks in Africa [1]. Dendritic cells (DCs) and macrophages are the main initial targets of EBOV infection [2]–[4]. A series of studies have shown that EBOV inhibits the production of type I IFN by these cells, while stimulating them to release large quantities of proinflammatory cytokines [5]–[7]. As a result, the virus spreads rapidly to cause an intense systemic inflammatory syndrome resembling septic shock [8]. The impaired innate immunity might also inhibit subsequent adaptive responses [5]–[7],[9]. A series of reports indicate that EBOV selectively weakens production of type I interferons (IFNs), while allowing production of other proinflammatory cytokines [5]–[7]. Epidemiological and animal studies support the idea that type I IFNs play a protective role against EBOV infection. Immunocompetent mice, which are resistant to infection with wild-type EBOV, become lethally infected when treated with antibody to type I IFN [10]. Moreover, IFNα production correlates with increased resistance in infected mice [11]. Further, administration of type I IFNs confers partial protection against EBOV infected monkeys [12]. Although type I IFNs were shown to be produced upon lethal EBOV infection in an animal model study, a study during an outbreak of Ebola hemorrhagic fever showed that IFNα levels were significantly higher in surviving patients than those with fatal infection [5],[6].

Two EBOV proteins, VP24 and VP35, are responsible for the suppression of type I IFN production [7], [13]–[15]. VP24 inhibits the cellular response to exogenous IFN by interacting with karyopherin α1, preventing the nuclear accumulation of tyrosine-phosphorylated Stat1 and Stat2 [15],[16]. VP35, on the other hand, has been shown to inhibit the activation of the transcription factor IRF3 by binding to dsRNA and inhibiting retinoic acid induced gene-I (RIG-I) signaling [13],[14],[17]. VP35 is also reported to interfere with the activation of the dsRNA-binding kinase, PKR [18]. However, an EBOV variant that was attenuated as a result of a point mutation in the VP35 RNA-binding domain was still capable of inhibiting IFNβ induction, suggesting the existence of another inhibitory mechanism [17],[19],[20]. Pertinent to this issue, Prins, et al., recently reported that VP35 impairs the activity of kinases important for IRF3 activation [21].

Although studies of VP35-mediated IFN antagonism have so far focused on the inhibition of IRF3, it has been demonstrated that a different transcription factor, IRF7, is largely responsible for the induction of type I IFN after virus infection, as evidenced by the abrogation of IFN production in *Irf7* −/− mice, but not in *Irf3* −/− mice [22]–[24]. IRF7, although similar to IRF3 in structure, differs in its expression and its mode of action [22],[25],[26]. The dominant role that IRF7 plays in IFN production in DCs has also been established: plasmacytoid DCs (pDCs), which produce the largest amounts of type I IFNs, express IRF7 at high levels, and its expression is further enhanced by IFN produced by positive feedback [23], [27]–[29]. In light of this and the evidence that DCs are a primary site of early EBOV infection, it seems important to investigate the mechanism of VP35's IFN antagonism in DCs, focusing on its effects on IRF7.

DCs are key players of innate immunity [30],[31]. Distributed widely in the body, DCs are among the first cells to recognize pathogen signals through toll-like receptors (TLRs) and other receptors [32]. In response, they produce large amounts of type I IFNs [27],[33], which in turn stimulate DC maturation to establish host resistance and facilitate the initiation of adaptive immune responses. The importance of studying EBOV infection of DCs gains additional urgency given the reports that some aspects of TLR signaling and pathogen processing in DCs are distinct from those in other cells [34]. For example, the RIG-I system is shown to be dispensable for IFN production in pDCs [35]. Moreover, type I IFN production in DCs involves another transcription factor, IRF8, that acts uniquely in the second phase of IFN transcription in DCs [36].

Here we report that VP35 potently inhibits type I IFN induction in mouse pDCs and other conventional (c)DCs in response to virus infection or TLR signaling, without inhibiting NF-κB activation. A yeast two-hybrid screen and co-immunoprecipitation analysis showed that VP35 interacts with IRF7 and IRF3 as well as two other cellular proteins, PIAS1 and Ubc9. The latter proteins are involved in the small ubiquitin-like modifier (SUMO) conjugation cascade [37]–[39]. SUMO proteins (SUMO1 through SUMO4) are composed of ∼100 amino acids, conserved from yeast to humans. They are covalently conjugated to a variety of proteins through lysine residues in a reversible manner, modulating their activities. SUMO modification affects many cellular processes, including stress response, transcription and protein-protein interactions. Similar to ubiquitination, the SUMO modification requires three step enzymatic reactions involving the E1 enzymes, Ubc9, an E2 enzyme and E3 ligases such as PIAS family proteins.

We show that VP35 augments SUMOylation of IRF7, leading to increased inhibition of IFN transcription by IRF7 that is at least in part mediated by PIAS1. A similar effect of VP35 was noted for IRF3. Supporting the view that SUMOylation is involved in the IFN transcription, we recently reported that IRF3 and IRF7 are modified by SUMO1 through SUMO3 in fibroblasts after viral infection. In that report SUMO molecules were covalently conjugated to IRF3/7 through TLR and RIG-I signaling which was linked to reduced IFN transcription, indicating that SUMO modification of IRF3/7 is a part of the negative feedback loop of normal IFN signaling [40]. Our results illustrates that VP35 makes use of this cellular mechanism to weaken host innate immunity.

## Results

### VP35 from wild-type and mouse-adapted EBOV inhibits type I IFN production in DCs

**Figure 1:**
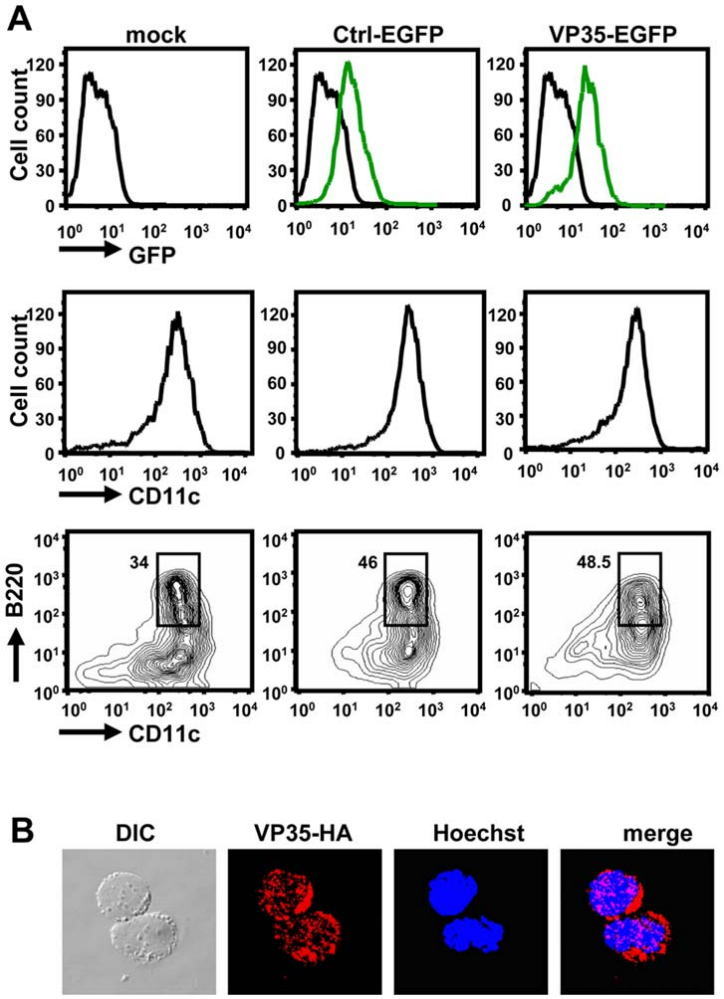
Expression of VP35 in pDCs and cDCs. A: BMDCs were transduced with pMSCV vector for VP35-EGFP or free EGFP (Ctrl-EGFP) on day 2 and allowed to develop in Flt3L for a total of 8 days. EGFP signals and expression of CD11c and B220 were monitored by flow cytometry. The bracketed areas indicate pDC population and the numbers represent the percentages in the total DC population. B: DCs were transduced with pMSCV vector for VP35-HA. On day 8 the cells were fixed, immunostained with antibody for HA followed by counterstaining with Hoechst for DNA. On the left is an image by differential interference contrast (DIC).

VP35 derived from a mouse-adapted EBOV variant was tagged with the enhanced green fluorescent protein (EGFP) or hemagglutinin (HA), cloned in the pMSCV retroviral vector, and introduced into bone marrow (BM)-derived DCs cultured in the presence of fms-like tyrosine kinase 3 ligand (Flt3L) [36],[41]. In the presence of Flt3L, all four major DC subsets are generated [30],[41]. Flow cytometry data in Figure 1A (upper panel) showed that EGFP-tagged VP35 (VP35-EGFP) was expressed in essentially all BMDCs in the culture, showing similar fluorescent intensity as cells expressing free EGFP. As shown in Figure 1A (middle and bottom panel), introduction of VP35 vector did not inhibit the generation of DCs, as verified by the expression of CD11c, the pan-DC marker on the cells transduced with VP35-EGFP, EGFP alone or mock transduced. Furthermore, the percentage of pDCs, as assessed by the B220 marker, was similar among these cells (between 35% and 50%), indicating that VP35 did not affect the ratio of pDCs and cDCs. Immunostaining analysis in Figure 1B showed that HA-tagged VP35 (VP35-HA) was present largely in the cytoplasm, consistent with the predominantly cytoplasmic localization of VP35 reported earlier [14]. Thus, VP35 can be efficiently expressed in BMDCs without inhibiting their development. The mouse-adapted VP35 differs from that of the wild-type Zaire EBOV in one amino acid (position 12, substituting V for A). We also constructed a vector for EGFP-tagged VP35 from the wild-type EBOV and found that this VP35 was expressed in a manner similar to the mouse adapted VP35 and its expression also did not interfere with the DC development (see below). Both vectors expressed the VP35 proteins of expected molecular mass, as judged by immunoblot analysis (Figure S1A).

**Figure 2:**
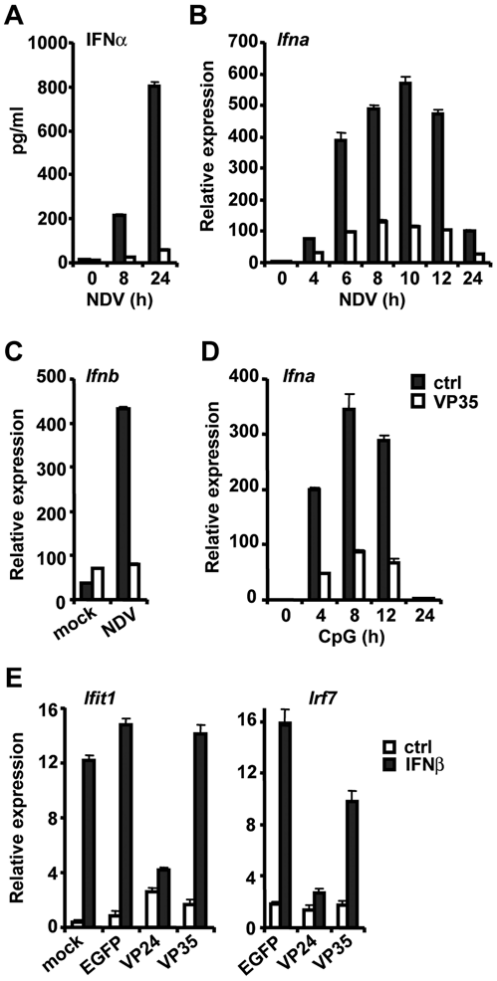
VP35 inhibits induction of IFNα and IFNβ in DCs. A and B: DCs transduced with VP35-EGFP (VP35, open bar) or free GFP (ctrl, solid bar) were infected with NDV for indicated times. IFNα protein production (A) and Ifnα transcript expression (B) were monitored by ELISA and qRT-PCR, respectively. Values in these experiments (and all below) represent the average of three determinations +/−S.D. C: DCs transduced as above were infected with NDV for 5 h or mock infected and tested for Ifnβ transcripts as in B. D: DCs transduced as above were stimulated with CpG DNA (1 µg/ml) for indicated times and Ifnα transcripts were measured as in B. E: DCs transduced as above or with VP24-EGFP were stimulated with IFNβ (100 U/ml) (solid bar) or vehicle (open bar) for 8 h and expression of Ifit1 and Irf7 transcripts was monitored by qRT-PCR.

Induction of type I IFNs was then tested in these DCs following infection with the Newcastle Disease virus (NDV). We have previously shown that both pDCs and cDCs produce high levels of type I IFNs after NDV infection [36]. In Figure 2A, we examined IFNα protein production in DCs expressing VP35-EGFP. NDV infection led to high IFNα production in control DCs expressing free EGFP, whereas little IFNα was produced in DCs expressing VP35-EGFP. Paralleling these results, NDV infection stimulated robust IFNα transcript expression in control DCs, but the expression was very meager in VP35-EGFP expressing DCs (Figure 2B). Similarly, NDV infection stimulated IFNβ transcript induction in control DCs, but it failed to do so in DCs expressing VP35-EGFP (Figure 2C). Since DCs produce type I IFNs in response to multiple toll-like receptor (TLR) signaling, including TLR9, we tested the effect of VP35-EGFP on CpG DNA-induced IFN transcription [32],[33]. In Figure 2D, VP35 strongly inhibited IFNα induction by CpG, supporting the idea that VP35 can inhibit type I IFN induction independently of dsRNA binding activity.

Both the mouse-adapted and wild-type VP35 proteins inhibited IFN induction in DCs after NDV and CpG stimulation (Figure 2A–D, Figure S1B, S1C). Thus, all studies presented in the remainder of this paper were conducted with the mouse-adapted VP35. It is of note that VP35-EGFP, VP35-HA and VP35 without a tag equally inhibited IFN induction (see below).

The complete induction of IFN in DCs involves two steps: initial transcription is triggered by IRF7, while the second round of transcription is induced by the IFN feedback response [27],[36]. If VP35 inhibits IFN transcription in the feedback phase, it would therefore inhibit the expression of other IFN stimulated genes as well. As shown in Figure 2E, VP35-EGFP did not inhibit expression of Ifit1, a typical IFN stimulated gene, and only modestly inhibited IRF7 induction. In contrast, other investigators have shown that EBOV VP24-EGFP, known to inhibit IFN stimulated transcription, strongly inhibited expression of these genes [15],[16]. These data indicate that VP35 inhibits the initial phase of IFN transcription in DCs.

### VP35 does not inhibit NF-κB activation

**Figure 3:**
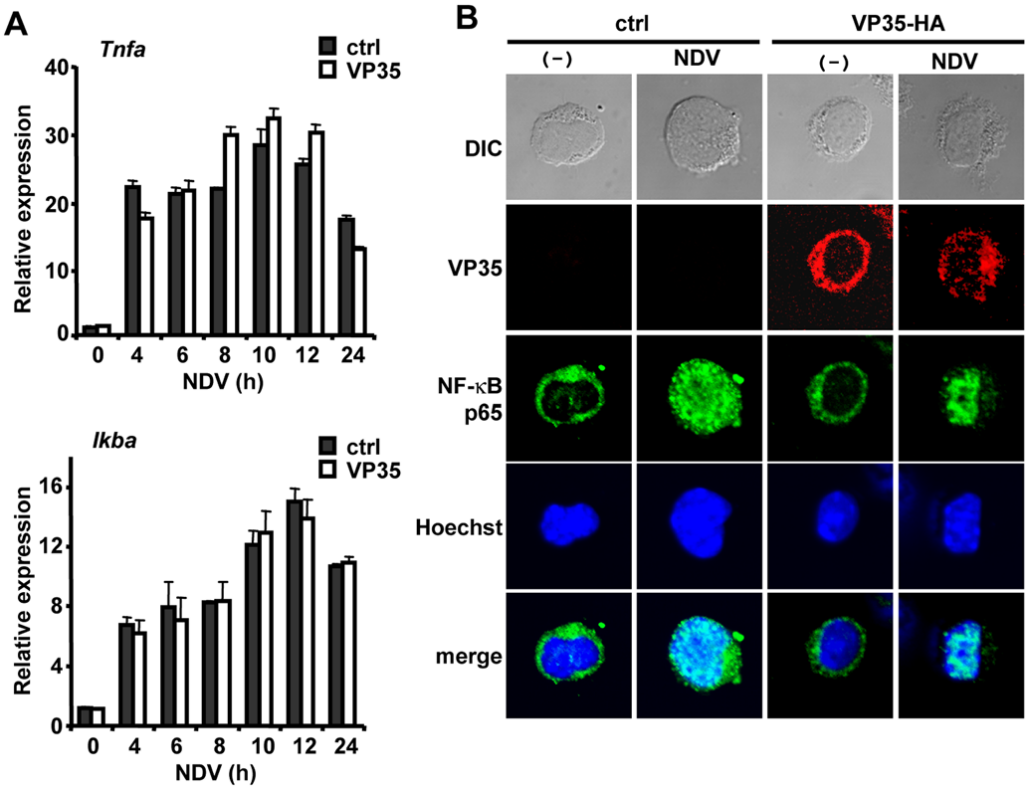
VP35 does not inhibit NF-κB activation. A: DCs transduced with VP35-EGFP (VP35) or free GFP (ctrl) were infected with NDV for indicated times and expression of Tnfα and Ikbα transcripts was monitored as in Figure 2B. B: DCs transduced with VP35-HA or empty vector (ctrl) were infected with NDV for 5 h and immunostained for HA or p65/RelA (NF-κB). About 200 DCs in three different fields were inspected to quantify DCs showing p65 nuclear translocation.

Pathogen signaling activates two separate transcription pathways involving IRF3/7 and NF-κB [32]. The former stimulates transcription of IFNαs, while the latter triggers that of proinflammatory cytokines, although both IRF3/7 and NF-κB are involved in IFNβ transcription [22],[32],[42]. We sought to assess the role of VP35 in the activation of NF-κB, considering that EBOV impairs type I IFN production, while often enhancing the production of other proinflammatory cytokines triggered by NF-κB [6],[7]. As seen in Figure 3A, NDV infection stimulated the expression of typical NF-κB targets, TNFα and IκBα, equally well in control and VP35-EGFP expressing DCs [42]. These data suggest that VP35 inhibits IRF3/7 dependent transcription without affecting NF-κB mediated transcription in DCs. To further assess the effect of VP35 on NF-κB activation, we looked for the nuclear translocation of p65/RelA, the major activating component of NF-κB [43]. Immunostaining data in Figure 3B showed that before NDV stimulation, p65/RelA was predominantly in the cytoplasm, but upon stimulation the majority of p65/relA translocated into the nucleus, both in control and VP35-HA expressing DCs. Of ∼200 stimulated DCs inspected, more than 85% displayed p65/RelA in the nucleus, irrespective of VP35-HA expression. These data support the idea that VP35 does not inhibit NF-κB activation in DCs.

### VP35 inhibits the recruitment of IRF7 to type I IFN genes in DCs

**Figure 4:**
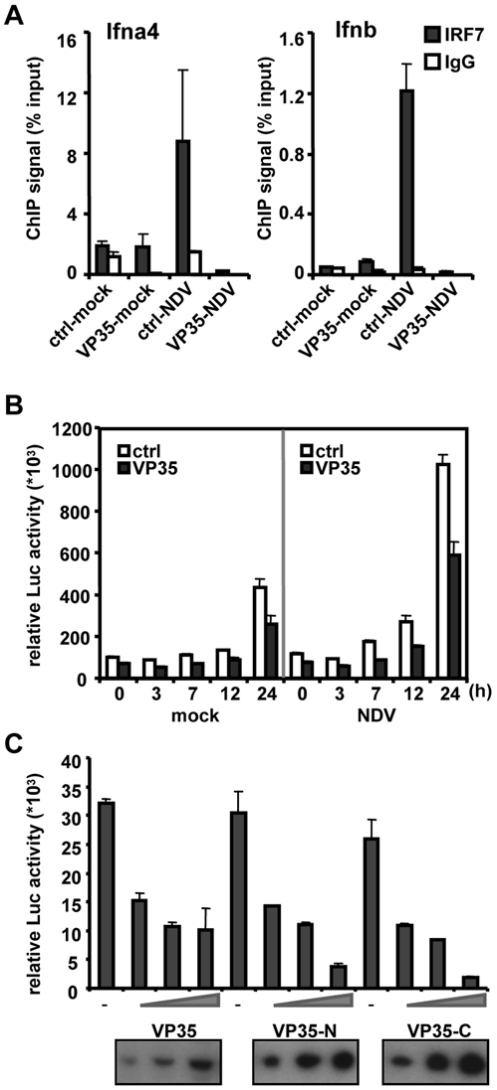
VP35 blocks recruitment of IRF7 to the *Ifna* and *Ifnb* genes in DCs. A: DCs transduced with VP35-HA or with control vector were infected with NDV for 7 h and chromatin was precipitated with anti-IRF7 antibody (solid bar) or normal rabbit IgG (open bar). Precipitated DNA was amplified for the *Ifna4* and *Ifnb* promoters by q-PCR. ChIP signals are expressed as the percentage of input DNA (1%). B: 293T cells (1×10^5^) were transfected with pcDNA3.1 vector for IRF7 (0.05 µg), VP35-HA (VP35) or empty vector (ctrl) (0.2 µg), along with IFNβ luciferase reporter (0.4 µg) and pRL-TK control reporter (0.01 µg) for 24 h, and infected with NDV for 24 h. Reporter activity was monitored by dual luciferase reporter assay. C: A549 cells (1×10^5^) transfected with the vector for Flag-IRF7 (0.02 µg), increasing amounts of VP35-HA (0.2–1 µg) and IFNβ reporter (0.4 µg) plus pRL-TK (0.01 µg) as above were infected with NDV for 24 h and reporter activity was measured as in B. Expression of VP35-HA was verified by immunoblot analysis in the bottom.

To ascertain whether VP35 inhibits IFN production by disabling IRF7, immunoprecipitation (ChIP) assay was carried out to examine the binding of IRF7 to the *Ifn* genes in DCs. Chromatin from control and VP35-HA-expressing DCs was precipitated with anti-IRF7 antibody, and precipitated DNA was tested for the *Ifna4* and *Ifnb* genes by quantitative (q) PCR [36]. In control DCs, IRF7 bound to both the *Ifna4* and *Ifnb* genes after NDV infection, but not after mock infection (Figure 4A). In contrast, little IRF7 binding was detected in VP35-HA-expressing DCs with or without NDV infection. In both cases, control IgG gave signals at background levels. These results indicate that VP35 blocks virus-induced recruitment of IRF7 to *Ifn* genes. Further supporting inhibition of IRF7 recruitment, VP35 from the wild-type EBOV similarly blocked NDV triggered IRF7 recruitment in these DCs (Figure S1D).

### VP35 inhibits IRF7-dependent IFNβ promoter activity

**Figure 5:**
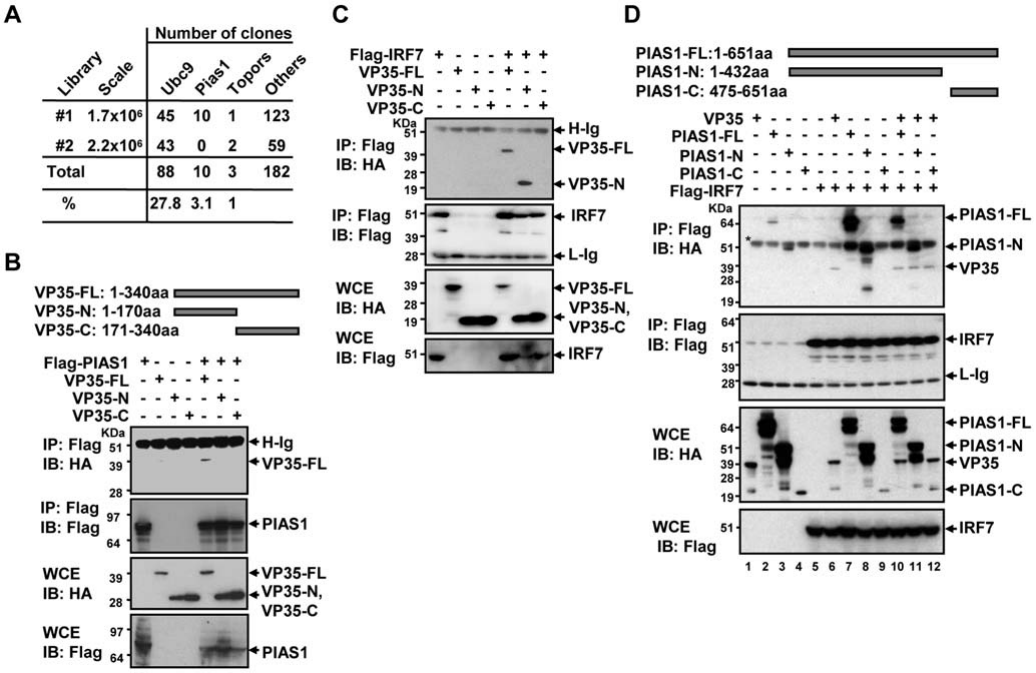
Interaction of VP35 with PIAS1 and IRF7. A: A summary of yeast two-hybrid screen. Two libraries from NDV-stimulated DCs were screened with full length VP35 as a bait. The numbers of sequenced clones are shown. B: 293T cells (1×10^6^) were transfected with pcDNA3.1 vector for full length Flag-PIAS1, or empty vector (2 µg) along with full length VP35-HA (VP35-FL) or truncated versions (2 µg each), for 30 h (see a truncation map on top). Extracts were precipitated by anti-Flag antibody and immunoblotted with anti-HA antibody (top gel). Whole cell extracts (WCE) were blotted with antibody to HA or Flag in the lower gels. C: Cells were transfected with indicated vector for Flag-IRF7 (2 µg) or full length or truncated VP35-HA (2 µg), and extracts were precipitated and blotted as in A. D: Interaction of VP35 with truncated PIAS1 and IRF7. Cells were transfected with HA-tagged full length PIAS1 (FL) (2 µg) or indicated truncations (see a PIAS1 truncation map on top) along with full length VP35-HA and Flag-IRF7 (2 µg each) and extracts were precipitated with anti-Flag antibody, blotted with anti-HA antibody. PIAS1-N migrated just below the Ig heavy chain (marked with *).

To further investigate VP35 inhibition of IRF7 function, IFNβ reporter assays were performed in 293T cells expressing IRF7 and VP35-HA (Figure 4B). As expected, transfection of IRF7 alone without VP35-HA enhanced IFNβ promoter activity even before infection, and NDV infection increased reporter activity by about two-fold. In both cases, cotransfection of VP35-HA inhibited IFNβ promoter activity by about 40%, suggesting that VP35 directly targets IRF7. In Figure 4C, VP35 truncations lacking the N-terminal or C-terminal half of VP35 (VP35-N and VP35-C) were tested for IFNβ promoter activity (see a VP35 truncation map in Figure 5B). Both truncations inhibited IFNβ promoter activity in a dose-dependent manner (see the bottom panel of Figure 4C for the levels of VP35 protein expression). The inhibition by VP-35C might have been expected, because dsRNA binding activity of VP35 resides in the C-terminal region [17]. These data indicate that the N- and C-terminal halves of VP35 both contribute to the inhibition of IRF7-mediated IFNβ promoter activity.

### Interaction of VP35 with the SUMOylation machinery: a yeast two-hybrid screen

The above data indicated that VP35 acts on a step downstream from pathogen signaling to disable the activity of IRF7 without affecting the activation of NF-κB. To gain a mechanistic clue for VP35 action, we searched for proteins that bind to VP35 by a yeast two-hybrid screen. cDNA libraries were constructed from NDV-stimulated DCs and screened with a full-length VP35 as a bait. As shown in Figure 5A, screening of two libraries yielded a number of clones implicated in the SUMO conjugation pathway, including Ubc9, the protein inhibitor of activated STAT (PIAS1), and Topors. Ubc9 is the sole E2 enzyme for SUMOylation, and PIAS1 is a SUMO E3 ligase important for IFN signaling [37],[38],[44],[45]. Topors also acts as a SUMO E3 ligase for some substrates [46]. These results pointed to a link between VP35 and the host cell SUMO conjugation machinery.

### VP35 interacts with both PIAS1 and IRF7

To further study a potential connection between VP35 and the SUMOylation machinery, co-immunoprecpitation (Co-IP) analysis was performed using 293T cells expressing Flag-tagged PIAS1 (Flag-PIAS1) and VP35-HA. In Figure 5B, Flag-PIAS1 coprecipitated full-length VP35, but neither of the truncated forms of VP35. Immunoblot analysis of whole cell extracts (WCE) showed that PIAS1 and VP35 were properly expressed in transfected cells. These data indicate that VP35 interacts with PIAS1, for which both the N- and C-terminal regions are required. We next asked if VP35 could bind to IRF7. As seen in Figure 5C, Flag-IRF7 indeed coprecipitated full-length VP35, demonstrating a direct VP35-IRF7 interaction. Further, Flag-IRF7 precipitated VP35-N, but not VP35-C, indicating that VP35-IRF7 interaction is mediated by the N-terminal half of VP35. Similar Co-IP experiments found that VP35 interacted with IRF7 before and after NDV infection, showing that VP35 interacts with both the constitutive and activated forms of IRF7 (Figure S2A).

The above results suggested the possibility that VP35 interacts with both PIAS1 and IRF7 to form a larger complex. To test this possibility, co-IP experiments were performed with cells expressing all three proteins. In Figure 5D, Flag-IRF7 precipitated PIAS1 in the absence of VP35, while it also precipitated VP35 in the absence of PIAS1 (lane 6, 7), indicating that IRF7 can interact with either PIAS1 or VP35, independently of the other protein. Furthermore, Flag-IRF7 co-precipitated both VP35 and PIAS1 when they were co-expressed (lane10). These data support the idea that VP35 could interact with PIAS1 and IRF7 simultaneously by forming a larger complex. Although there seemed a slight reduction in the amount of precipitated PIAS1 in the presence of VP35 (lane 7 vs. 10), multiple other experiments showed similar amounts of PIAS1 precipitated with and without VP35, supporting again that the three proteins interact with each other without competition. Co-IP analysis of PIAS1 deletions, also shown in Figure 5D, indicated that the N-terminal region of PIAS1 is important for the interaction with IRF7 (lane 8, 9, 11, 12). In addition, we tested a series of IRF7 deletions and found that IRF7 binds to VP35 through the two regions in the C-terminal domain predicted to juxtapose in a 3D structure analysis [47] (Figure S2B, S2C).

### PIAS1 conjugates SUMO onto IRF7 and inhibits IFNβ promoter activity

**Figure 6:**
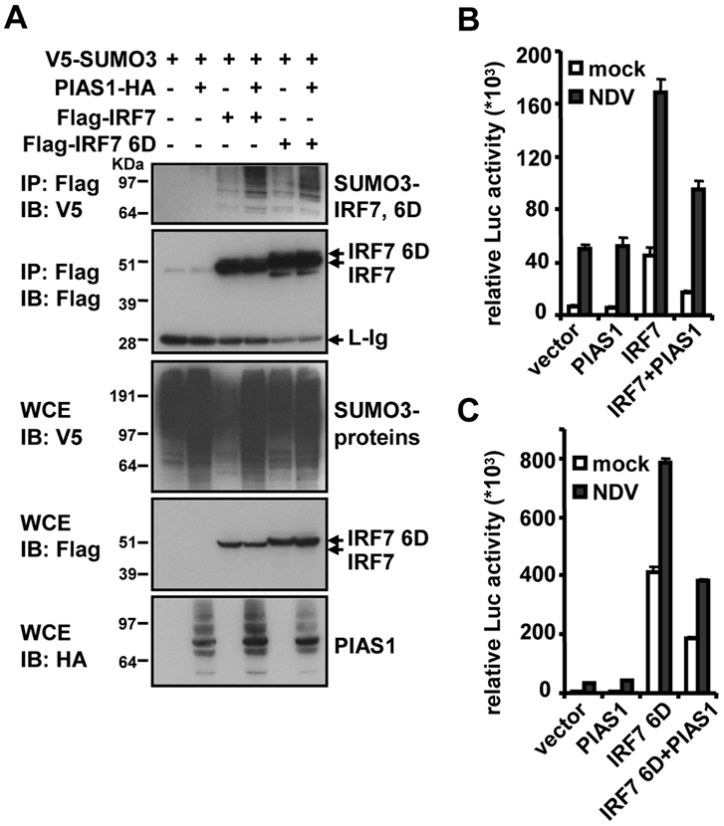
SUMOylation of IRF7 by PIAS1. A: 293T cells (1×10^6^) were transfected with V5-tagged SUMO3 (0.5 µg), PIAS1-HA (2 µg) along with Flag-IRF7 or Flag-IRF7 6D (1 µg) for 30 h. Extracts were precipitated with antibody to Flag and blotted with antibody to V5. B: Cells were transfected with PIAS1-HA (0.2 µg) or wild type IRF7 alone (0.02 µg) or together, along with IFNβ reporter plus pRL-TK for 24 h followed by NDV infection for 24 h. Luciferase activity was measured as in Figure 4B. C: Above experiments were performed with IRF7 6D in place of wild type IRF7.

The three-way interactions seen above, combined with extensive reports linking SUMO modifications to transcriptional repression, pointed to the possibility that VP35 represses IRF7-mediated transcription through SUMO conjugation [37],[38],[48]. To test this possibility, we asked whether PIAS1 could SUMOylate IRF7. Cells expressing V5-tagged SUMO3 and PIAS1-HA along with Flag-IRF7 were immunoprecipitated with anti-Flag antibody and tested for SUMOylation by immunoblot analysis. When coexpressed with PIAS1, IRF7 immune precipitates displayed extensive SUMO conjugation (see the slow migrating bands above 64 KDa, Figure 6A, upper panel). In the absence of PIAS1, however, IRF7 precipitates showed only meager SUMO conjugation, indicating that PIAS1 indeed mediated IRF7 SUMOylation. To assess whether PIAS1 could SUMOylate an activated form of IRF7, we tested a constitutively active IRF7, called 6D, in which six serine residues in the C-terminal domain were replaced with phosphomemic aspartic acids [49]. The IRF7 6D was also SUMOylated by PIAS1 in a manner similar to wild type. Immunoblot analysis of whole cell extracts showed that many proteins were broadly SUMOylated, irrespective of PIAS1 and IRF7 transfection, further supporting the specificity of PIAS1-dependent IRF7 SUMOylation (Figure 6A, middle panel). Multiple SUMO-conjugated bands found in the IRF7 precipitates may be attributed to conjugation of polymeric SUMO chains, although covalent binding of other peptides is another possibility [50],[51]. We found that under similar conditions, PIAS1 also conjugated SUMO1 onto IRF7, although less robustly than SUMO3, in line with our previous report and those indicating that different SUMO molecules can be conjugated to a single protein (Figure S3) [38],[40],[52]. To our knowledge, an E3 ligase for IRF7 has not been identified before, and this is the first report to show that PIAS1 functions as an enzyme catalyzing IRF7 SUMOylation.

Given that SUMOylation is linked to transcriptional repression, we then examined whether PIAS1 represses activity of IRF7 in IFNβ promoter activity. In Figure 6B, constitutive and NDV-stimulated IFNβ reporter activity was strongly enhanced by transfection of IRF7, as expected [23],[40]. However, cotransfection of PIAS1 led to ∼50% reduction in the reporter activity. As seen in Figure 6C, IRF7 6D led to even greater enhancement than wild type IRF7 in IFNβ promoter activity, which was again repressed by ∼50% upon co-transfection of PIAS1. These data indicate that PIAS1 represses IRF7's transcriptional activity, consistent with the previous reports that PIAS1 negatively regulates the activity of several transcription factors [44],[45],[53],[54].

### VP35 promotes PIAS1-mediated IRF7 SUMOylation

**Figure 7:**
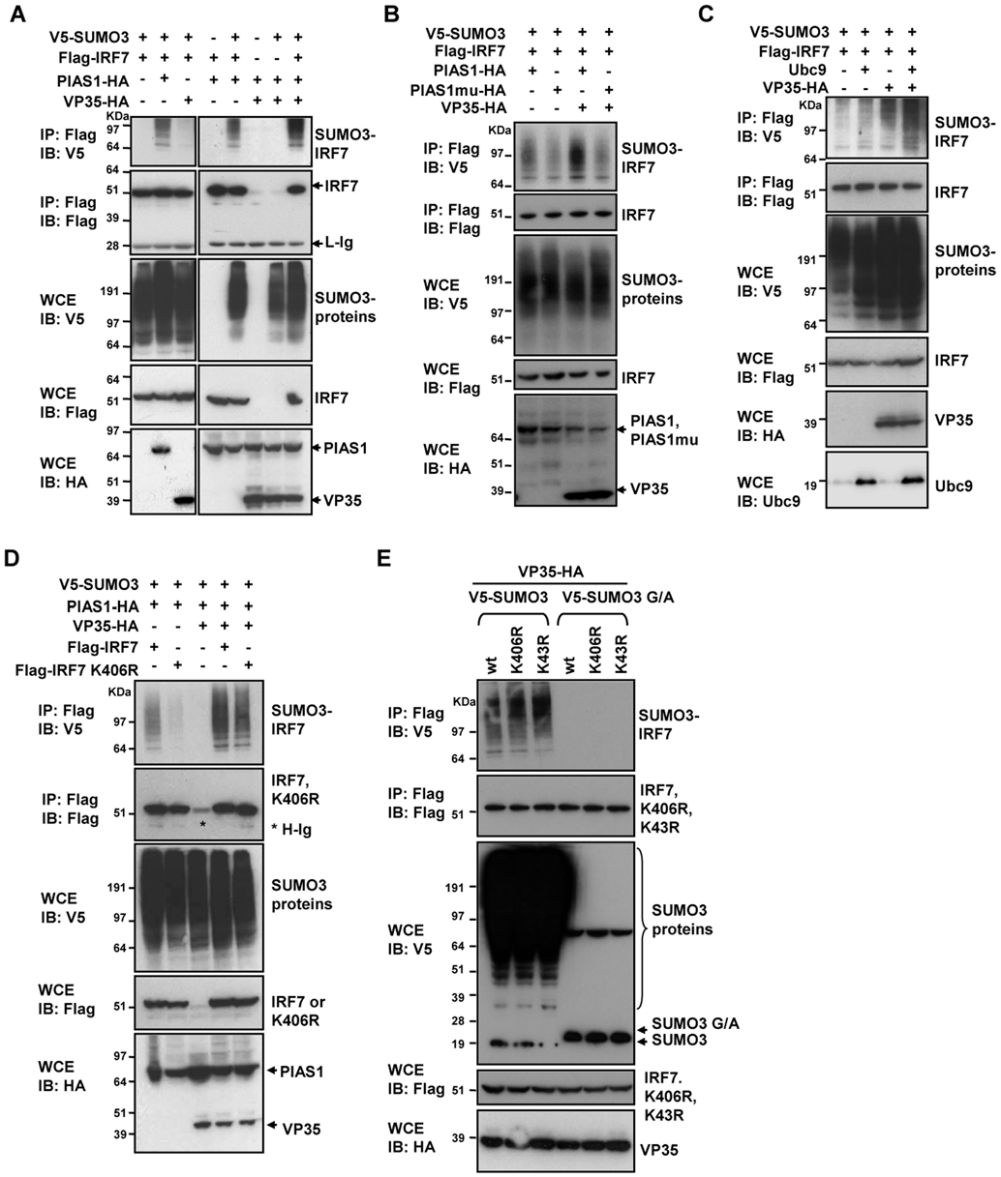
VP35 increases PIAS1 mediated IRF7 SUMOylation. A: 293T cells (1×10^6^) were transfected with V5-tagged SUMO3 (0.5 µg), Flag-IRF7 (1 µg) along with PIAS1-HA (1 µg) and VP35-HA (2 µg) for 30 h (top panel). Extracts were precipitated with anti-Flag antibody and blotted with anti-V5 antibody. Whole cell extracts were blotted with indicated antibodies (lower panels). B: Cells were transfected with V5-tagged SUMO3 and Flag-IRF7 as above, along with wild type PIAS1-HA or a PIAS1 mutant (PIAS1mu) (1 µg) and extracts were precipitated with anti-Flag antibody and blotted with indicated antibodies. C: Cells were transfected with V5-tagged SUMO3 and Flag-IRF7 as above, along with Ubc9 (1 µg) and extracts were precipitated with anti-Flag antibody and blotted with indicated antibodies. D: 293T cells were transfected with V5-tagged SUMO3 (0.5 µg), Flag-wild type IRF7 or the K406R mutant (1 µg) along with PIAS1-HA (1 µg) and VP35-HA (2 µg) (top panel). Extracts were precipitated with anti-Flag antibody and blotted with anti-V5 antibody. Whole cell extracts were blotted with indicated antibodies (lower panels). E: Cells were transfected with V5-tagged wild type SUMO3 or a conjugation-defective SUMO3 mutant (SUMO3 G/A, see a diagram on the right) (0.5 µg), Flag-tagged wild type IRF7, K406R, or K43R (1 µg) and VP35-HA (2 µg) for 30 h (top panel). Extracts were precipitated with anti-Flag antibody and blotted with anti-V5 antibody. Whole cell extracts were blotted with indicated antibodies (lower panels).

We next tested the effect of VP35 on PIAS1-mediated IRF7 SUMOylation. Cells expressing V5-SUMO3, Flag-IRF7, VP35-HA, and PIAS1-HA were immunoprecipitated with anti-Flag antibody and tested for SUMO conjugation by anti-V5 antibody (Figure 7A upper panel). IRF7 immune precipitates showed increased SUMO conjugation in the presence of PIAS1. In the presence of VP35, in contrast, IRF7 precipitates showed little increase in SUMOylation under these conditions, indicating that PIAS1, but not VP35 acted as a SUMO ligase for IRF7. Importantly, in the presence of both PIAS1 and VP35, the levels of SUMO conjugated IRF7 were significantly greater than those expressing PIAS1 alone. Analysis of whole cell extracts showed that exogenously expressed proteins were properly expressed in these cells (Figure 7A, lower panel). In agreement with the idea that VP35 promotes IRF7 SUMOylation, VP35, when expressed at higher levels, increased IRF7 SUMOylation in a dose dependent manner even in the absence of PIAS1 (Figure S4A).

To further assess the ability of VP35 to increase PIAS1-mediated IRF7 SUMOylation, we tested a PIAS1 mutant that has a substitution within the catalytic domain (PIAS1mu-HA) [55]. As seen in Figure 7B, IRF7 was only minimally SUMOylated in the presence of this mutant, unlike extensive SUMOylation observed by the wild type PIAS1. Addition of VP35 increased the extent of SUMOylation by the wild type PIAS1, as expected. In contrast, there was no discernible increase in IRF7 SUMOylation by the PIAS1 mutant. These data indicate that VP35 indeed promotes PIAS1-mediated SUMOylation of IRF7. Since Ubc9 was found to interact with VP35 in our yeast two-hybrid screen, we next tested if VP35 also promotes IRF7 SUMOylation. Results in Figure 7C show that while transfection of Ubc9 or VP35 alone increased IRF7 SUMOylation, addition of both Ubc9 and VP35 augmented the level of IRF7 SUMOylation.

To further substantiate the involvement of VP35 in IRF7 SUMOylation, we asked if it increases SUMO conjugation at the previously identified single SUMO site, the lysine (K) reside at 406 [40]. As presented in Figure 7D, in the absence of VP35, wild type IRF7 was efficiently SUMOylated by PIAS1, but not the K406R mutant. However, in the presence of VP35, the K406R mutant still showed SUMO conjugation, although less extensively than wild type IRF7. Another potential SUMO conjugation site at K43 in IRF7, when mutated did not eliminate IRF7 SUMOylation in the presence of VP35 (Figure S4B). These data indicate that VP35 promotes conjugation of SUMO molecules at multiple sites in IRF7. To verify that the IRF7 bands reacted with antibody to V5 (tagged to SUMO3) were indeed products of SUMOylation, we tested wild type SUMO3 and the conjugation defective SUMO3 (SUMO3 G/A) in the SUMOylation assay. Data in Figure 7E showed that wild type SUMO, but not the mutant produced SUMO-conjugated bands for both wild type and mutant IRF7. In line with the view that VP35 triggers IRF7 SUMOylation at multiple K resides, a recent analysis has expanded potential SUMOylation sites beyond the those predicted by previous models based on the ΨKXE motif [56]. According to the new model, ten additional K residues in IRF7 can potentially be SUMOylated (Table S1). Nevertheless, given that the K406R mutant was less efficiently SUMOylated than wild type IRF7 in the presence of VP35, it is likely that VP35 utilizes this site as well to increase IRF7 SUMOylation.

### VP35 enhances IRF3 SUMOylation

Although not indispensable, IRF3 plays a significant role in IFN transcription in various cell types except for pDCs [22],[23],[35]. In view of the fact that VP35 inhibits IRF3's ability to stimulate IFN transcription and that IRF3 is SUMOylated after viral infection, it was of interest to test if VP35 increases SUMOylation of IRF3 as well [13],[17],[21],[40]. As shown in Figure S5A, B,C, wild type IRF3 and IRF3 5D, a constitutively active form of IRF3 showed increased SUMOylation in the presence of VP35: IRF3 5D was SUMOylated to a greater extend than wild type IRF3. We also found that VP35 inhibited IFNβ promoter activity by both IRF3 and IRF3 5D under these conditions (Figure S5 D, E). These results are consistent with the idea that VP35 inhibits IFN transcription by promoting SUMOylation of both IRF3 and IRF7 before and after their activation.

### VP35 increases inhibition of IFNβ promoter activity by IRF7

**Figure 8:**
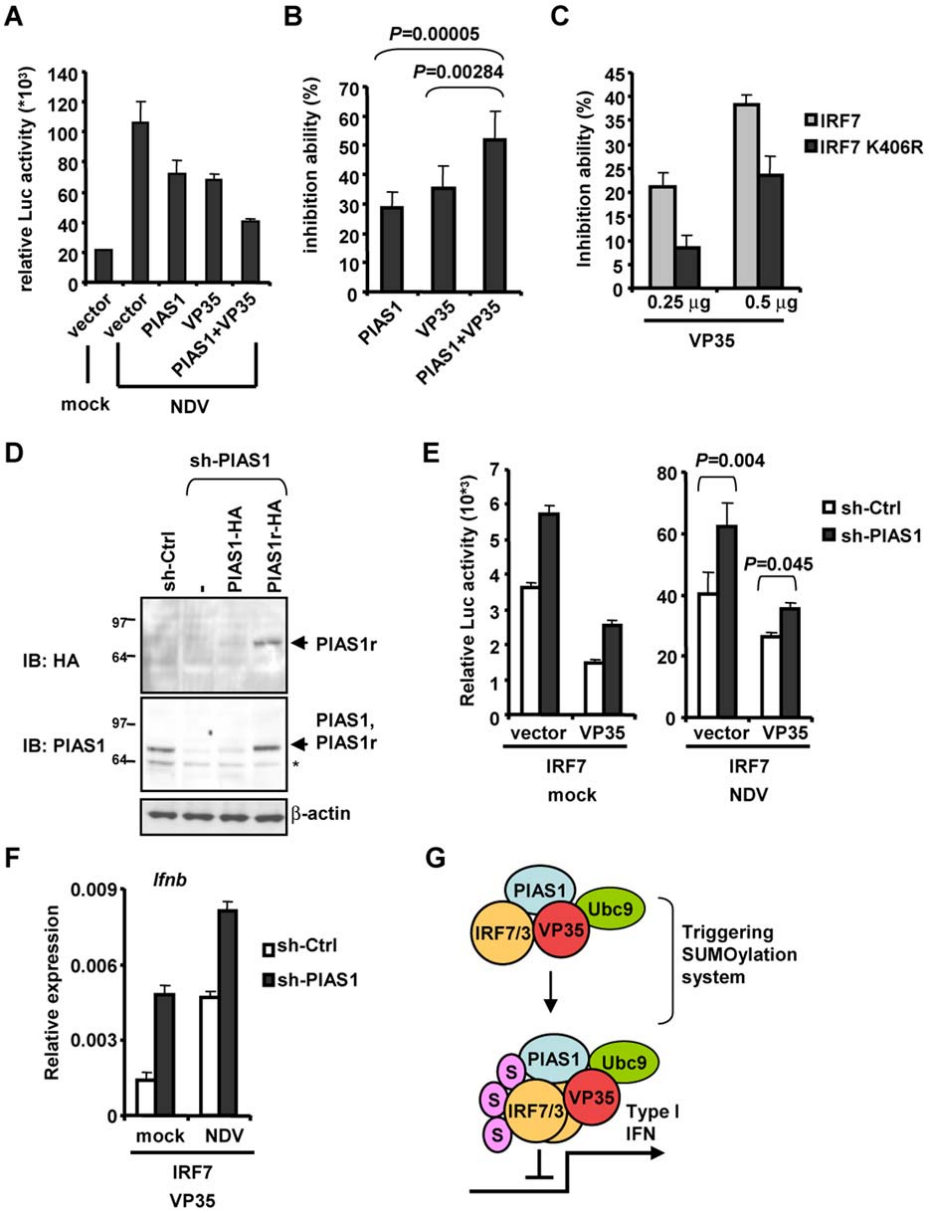
VP35 Increases IRF7 SUMOylation. A: Cells were transfected with PIAS1-HA or VP35-HA or both, a vector for IRF7 (0.02 µg each), and IFNβ reporter plus pRL-TK and stimulated with NDV for 24 h, and luciferase activity was measured as in Figure 4B (top panel). B: Experiments above were performed eight times each with triplicate determinations and levels of repression was averaged and quantified. C: Cells were transfected with two doses of VP35 with wild type IRF7 or IRF7 K406R (0.02 µg), along with IFNβ reporter plus pRL-TK for 24 h followed by NDV infection 24 h. Luciferase activity was measured as in Figure 4B. D: L929 cells (1×10^6^) transduced with control or PIAS1 shRNA retroviral vector. Some cells were also transduced with wild type PIAS1-HA or PIAS1r-HA vector that is resistant to PIAS1 shRNA. While cell extracts were blotted with anti-PIAS1 antibody. * denotes a nonspecific band. E: L929 cells (1×10^5^) transduced with control or PIAS1 shRNA vector were transfected with VP35-HA, IRF7 (0.02 µg each) and IFNβ reporter plus pRL-TK and with or without stimulation by NDV for 24 h, and luciferase activity was measured as in Figure 8A. F: NIH3T3 cells (1×10^5^) transduced with control or PIAS1 shRNA vector were further transduced with both of IRF7 and VP35 for 3 days. Cells were then infected with NDV for 24 h and IFNβ transcripts were measured in Figure 2C. G: A model for VP35 action. VP35 interacts with the host SUMOylation machinery, including Ubc9 and PIAS1, the SUMO E2 enzyme and E3 ligase, respectively. VP35 also interacts with IRF7 (and IRF3) bringing IRF7 (and IRF3) to the SUMOylation machinery, and promotes extensive SUMOylation of IRF7 (and IRF3). The premature SUMOylation of the IRFs abrogates their ability to activate IFN transcription causing diminished IFN production. It should be noted (i) VP35 has additional mechanisms of inhibiting IFN transcription and (ii) VP35 may involve other SUMO E3 ligases to increase IRF7 SUMOylation.

Because the combination of VP35 and PIAS1 increased IRF7 SUMOylation over that by each protein alone, it was of importance to test if VP35 and PIAS1 together would exacerbate the repression of IFNβ transcription. In Figure 8A, NDV-induced IFNβ promoter activity was reduced to the greater extent (61% inhibition) when PIAS1 and VP35 were both expressed, compared to when each protein was expressed alone (32 to 36% inhibition). To ascertain whether the combination of VP35 and PIAS1 results in greater inhibition of IFN transcription, similar assays were conducted eight times and levels of repression was quantified in Figure 8B. Again, the combination of the two proteins produced greater inhibition relative to the inhibition by each protein alone (P = 0.003 to 0.00005).

We next sought to evaluate the importance of SUMO conjugation at K406 in IRF7 in inhibition of IFNβ promoter activity, as SUMOylation of this residue appeared to be increased by VP35 (Figure 7D). As shown in Figure 8C, VP35 inhibited IFNβ reporter activity by the K406R mutant less robustly than that by wild type IRF7 and this effect was VP35 dose dependent (40% inhibition by K406R vs 70% inhibition by wild type IRF7). These data further support the view that VP35 inhibits IFN transcription by boosting IRF7 SUMOylation.

To further evaluate the role of PIAS1 in mediating VP35's inhibitory effect, we next tested whether PIAS1 knockdown could relieve VP35 inhibition of IFN reporter activity. A retroviral vector harboring PIAS1 shRNA reduced the expression of endogenous PIAS1 to an almost undetectable level in L292 cells. Moreover, expression of HA-tagged wild type PIAS1, but not a mutant PIAS1 resistant to the inhibitory effect of shRNA (PIAS1r-HA) was also knocked down by this shRNA vector (Figure 8D). As shown in Figure 8E (left panel), cells with PIAS1 shRNA showed higher IFNβ promoter activation by IRF7 (∼1.6 fold) compared to cells with control shRNA in the presence and absence of VP35. Likewise PIAS1 shRNA lessened VP35 inhibition of IFNβ promoter activity after NDV stimulation (Figure 8E right panel). In addition, in PIAS1 knockdown cells VP35 inhibited IRF7 stimulated IFNβ mRNA expression less well than in control shRNA cells, as noted by a ∼3 fold increase in transcript levels (Figure 8F). These results support the view that VP35 interacts with PIAS1 and IRF7 and promotes IRF7 SUMOylation, leading to efficient repression of IFNβ transcription, although our data do not exclude the possibility that VP35 may act on other SUMO ligases to inhibit IFN transcription.

## Discussion

Viruses employ diverse strategies to counter the antiviral activity of IFNs [57]. Some RNA and DNA viruses disable IRF3 or IRF7 by modulating ubiquitination processes, thereby hastening their degradation [22],[58],[59]. Some DNA viruses regulate cellular SUMOylation processes to increase their own infectivity. ICP0, a herpes simplex virus protein, inhibits SUMO modification of promyelocytic leukemia protein (PML), facilitating its degradation and the disruption of nuclear bodies [60]. The ICP0 mutation that eliminates this activity weakens lytic infection by the virus. Similarly, the E1E proteins of the cytomegalovirus inhibits SUMOylation of PML and SP100, leading to the disassembly of nuclear bodies [61]. Moreover, the adenoviral protein Gamp1 facilitates ubiquitination and degradation of SUMO E1 enzymes to inhibit global SUMOylation [62]. However, except for a few recent reports, SUMO modification by RNA viruses has not been extensively reported so far [40],[63].

This work began with the observations that VP35 potently inhibits type I IFN transcription in DCs, the cell type that produces much of the IFN in the body and that is a primary site of early EBOV infection. Subsequent studies of underlying mechanisms revealed that VP35 disables the activity of IRF7, the transcription factor essential for type I IFN induction, by making use of the cellular SUMOylation machinery.

### VP35 action in DCs

The inhibition of NDV-induced IFN transcription by VP35 in both pDCs and cDCs noted in this work is in agreement with previous investigations of VP35 activity in non-DCs [13],[17],[20]. Nevertheless, our results differ from those of previous reports in several important aspects. First, the effect of VP35 was previously ascribed to reduced activation of IRF3, a factor that has since been shown to be dispensable for IFN induction in various cell types including DCs [22],[23], whereas our study primarily focused on VP35 inhibition of IRF7, a factor known to be critically required for IFN transcription, particularly in DCs. In addition, the dsRNA-binding activity mapped to the C-terminal region of VP35 was previously proposed to be important for inhibition of IFN production, although these studies predicted the presence of an additional mechanism by which VP35 inhibits IFN production [17],[21]. We noted that VP35 inhibits CpG DNA-mediated IFN transcription, consistent with an inhibitory mechanism independent of dsRNA-binding activity. We also found that the N-terminal half of VP35 was required for inhibition of IFN transcription, in addition to the C-terminal half. The N-terminal VP35 was subsequently found essential for the interaction with IRF7 and PIAS1, through which VP35 inhibited IFN transcription. Thus, it seems reasonable to suggest that the enhanced SUMOylation of IRF7 by VP35 observed in this work represents the missing mechanism foreseen by earlier studies [17].

Another notable aspect of our findings is that while VP35 strongly inhibited IFN transcription, it only marginally affected NF-κB activation, as evidenced by intact IkBα induction and normal nuclear translocation of p65/RelA in VP35 expressing DCs. This result is interesting, since NF-κB is essential for the expression of many pro-inflammatory cytokines and chemokines [42],[43]. The selective abrogation of IRF7 activation, sparing NF-κB activation is reminiscent of the characteristic pathogenesis of EBOV infection, in which impaired IFN induction accompanies copious production of other proinflammatory cytokines [6],[7].

### VP35 exploits the cellular SUMOylation machinery to inhibit IRF7 activation

Results of yeast two-hybrid screens and the subsequent Co-IP experiments revealed a clear link between VP35 and SUMO modification: VP35 formed a complex with the SUMO ligase PIAS1 and IRF7, augmented PIAS1-mediated IRF7 SUMOylation, and increased the repression of IFN promoter activity (Diagram in Figure 8G). One can envisage that VP35, although not itself a SUMO E3 ligase, brings IRF7 to the cellular SUMO machinery, causing increased IRF7 SUMOylation and decreased IFN transcription. The observations that both the wild-type and constitutively active IRF7 were SUMOylated by PIAS1, and that VP35 inhibited activities of both forms of IRF7, indicate that VP35 can promote SUMOylation of IRF7 before and after activation. Further supporting a link between VP35 and SUMOylation, we noted that VP35 also increases SUMO conjugation of wild type and an active form of IRF3. The idea that VP35 makes use of the cellular SUMO system is further supported by our recent report that both IRF3 and IRF7 are SUMOylated following viral infection [40]. It is likely that IRF7 SUMOylation represents a feedback mechanism by which to attenuate IFN transcription post-activation, allowing cells to limit excessive inflammatory responses [64]. Our results are consistent with the view that VP35 prematurely causes extensive IRF7 SUMOylation (and that of IRF3 in some cells) to halt the transcriptional activation of *Ifn* genes. In that study, we showed that IRF7 is SUMOylated mainly at K406, leading to reduced IFN production. Our present analyses indicate that VP35 triggers in SUMOylation of not only this site but additional K residues.

In this paper we show that PIAS1 conjugates SUMO1 and SUMO3 to IRF7 and represses IRF7 dependent IFN transcription. To our knowledge, a SUMO E3 ligase for IRF7 has not been identified to date, and this is the first demonstration that PIAS1 serves as an E3 ligase for IRF7. We found that the combination of VP35 and PIAS1 exacerbated inhibition of IFN transcription, consistent with the idea that VP35 promotes IRF7 SUMOylation through PIAS1. This idea is further supported by the observation that this inhibition was relieved by PIAS1 shRNA. While our data point to a significant role for PIAS1 in VP35 mediated repression of IFN transcription, it is possible that VP35 mobilizes other ligases to achieve greatest inhibition of IFN induction. PIAS1 belongs to the PIAS family, which includes three additional members [65]. The founding member, PIAS1, inhibits STAT1 activation to block the expression of some, but not all IFN-responsive genes [45]. *Pias1* −/− macrophages are, thus, hypersensitive to IFN stimulation [45]. Although a previous report showed that PIAS1 inhibits the DNA-binding activity of STAT1 independent of SUMOylation, a more recent study showed that it also SUMOylates STAT1 [44],[54]. These and additional reports that PIAS family proteins conjugate SUMO molecules onto IRF1 and IRF2 appear to support a role for the PIAS family in regulating the IFN system [40],[51],[66].

A large body of literature illustrates a strong link between SUMOylation and transcriptional repression through multiple mechanisms [37],[38],[65]. For example, SUMO modification influences nuclear-cytoplasmic transport of a number of proteins, while some SUMOylated transcription factors repress transcription by interfering with their nuclear retention and/or export [37],[38]. SUMO-conjugated proteins may also be recruited to a region of repressed chromatin, as reported for the recruitment of SUMOylated homeodomain-interacting protein kinase 2 to nuclear bodies [67]. Similarly, SUMO-conjugated Sp3 is sequestered in nuclear bodies [68]. Finally, SUMO-modified proteins such as p300, ELK, and PPARγ recruit co-repressors and histone deacetylases to establish a repressive chromatin environment [37],[65],[69]. This mechanism may explain how SUMOylated proteins, which make up only a fraction of the total proteins, can cause transcriptional repression. At present it is uncertain exactly how SUMOylated IRF7 blocks IFN transcription, although it is clear that it disables recruitment to *Ifn* genes.

In summary, this work describes a viral strategy that exploits the host SUMOylation system to inactivate antiviral innate immunity. It will be of importance to elucidate the mechanism by which SUMO-modified IRF7 represses IFN gene transcription in DCs.

## Materials and Methods

### Plasmids and retroviral vectors

cDNAs encoding VP35 and VP24 of the mouse-adapted EBOV were generated by site directed mutagenesis from the pcDNA3.1 plasmids harboring VP35 and VP24 of the Zaire subtype EBOV using the QuikChange kit (Stratagene). cDNAs for VP35 and VP24 were cloned into appropriate plasmid vectors to fuse to the EGFP- or HA at the C-terminus and the fused cDNAs were then inserted into pMSCV-puro vector (Clontech). Viral supernatants were prepared from 293ET cells transfected with the above vectors, plus plasmids for VSV-G envelope and gag/pol. Mouse full-length PIAS1 was cloned from IFNβ-stimulated NIH3T3 cells and inserted into pcDNA3.1 with a Flag or HA tag. The HA-tagged PIAS1 mutant in which the cysteine at 351 was replaced by serine was constructed in pcDNA3.1 by site directed mutagenesis. SUMO3 cDNA was cloned in pcDNA3.1 with a V5-tag at the N-terminus. The V5-tagged SUMO3-G/A in pcDNA3.1 was constructed by replacing two glycines to alanines at aa 91 and 92 by site direct mutagenesis. All resultant constructs were sequenced to verify correct cloning. Expression vectors for mouse IRF7, IRF7 K406R, IRF7 K43R, IRF3 and IRF3 5D in pcDNA3.1 and the IFNβ promoter construct were described [40]. Deletion constructs for VP35, PIAS1 or IRF7 were prepared by standard cloning procedures. The PIAS1 shRNA retroviral vector was constructed in pSUPERretro vector (Oligoengine) by inserting gaaaccagttgtccacaagaa which targets nucleotide position 624–644 of mouse PIAS1. L929 or NIH3T3 cells were transduced with the shRNA retroviral vector or control shRNA vector essentially as described [70]. Briefly, cells were transduced with viral supernatants by spinoculation twice over two consecutive days and were selected by puromycin (2 µg/ml) for 3 days prior to use. Antibodies for Flag-conjugated to beads, (M2), HA, V5 and PIAS1 were obtained from Sigma, Roche, Invitrogen and Epitomics, respectively.

### BMDCs and viral transduction

All animal work performed under protocols approved the animal care and use committees of NICHD. BMDCs were generated in the presence of Flt3L from C57BL/6 mice as described [36],[71]. Two days following the initiation of culture, cells were transduced with pMSCV vectors for VP35-EGFP, VP35-HA, free EGFP, or without insert by two consecutive spinoculations. Cells were selected by 1 µg/ml puromycin for the remaining period. On day 7 or day 8, cells were infected with NDV (Heartz strain) at a MOI of 2 or stimulated with CpG (ODN1826, Invitrogen) or IFNβ (PBL) at indicated concentrations for indicated periods.

### Flow cytometry and immunostaining

To monitor DC surface markers, cells were incubated with Phycoerythrin-conjugated B220/CD45R and biotin conjugated antibodies against CD11c/HL3 followed by Streptavidin-Phycoerythrin-Cy5 (both from BD Pharmingen). Stained cells were analyzed on FACSCaliber (Becton Dickinson) and data were processed by the FlowJo software. For immunostaining, DCs were placed on cytospin slides and fixed with 4% paraformaldehyde, permeabilized with 0.5% Triton X-100 and stained with indicated antibodies as described [71]. To detect HA-tagged VP35, cells were stained with anti-HA antibody (Roche) followed by Alexa-Fluor 568 conjugated goat anti-rat IgG (Molecular Probes), counterstained with Hoechst 33342. Stained cells were viewed on a Leica Model TCS-SP2 confocal microscope.

### Quantitative RT-PCR

cDNA was synthesized from 0.5 µg of total RNA from indicated DCs using SuperScript II reverse transcriptase (Invitrogen). PCR amplification was performed with 4 ng of cDNA in 10 µl of SYBER Green PCR Master Mix (Applied Biosystems) with 3 µM of primers in the ABI prism 7500 fast Real-Time PCR System (Applied Biosystems). DC supernatants were tested for IFNα production using the mouse IFNα ELISA Kit (PBL).

### Yeast two-hybrid screen

Two cDNA libraries were constructed from DCs stimulated with NDV for 6 h in pDEST22 vector using the CloneMiner cDNA Library Construction Kit (Invitrogen) according to the manufacture's designated procedures. Both libraries had the average insert size of ∼1.5 kbp. 4.41×10^6^ yeast clones were screened with ProQuest two-hybrid system (Invitrogen) with the full length VP35 as a bait, and resultant 317 positive clones were sequenced.

### Coimmunoprecipitation, immunoblot and SUMO conjugation assay

293T cells (1×10^6^) were transfected with indicated expression vectors for 30 h, extracts were prepared in 500 µl lysis buffer (1% NP40, 50 mM Tris-HCl [pH 7.5], 150 mM NaCl, 2.7 mM KCl). Four hundred µl of lysates were incubated with anti-Flag antibody beads (Sigma) for overnight, and precipitates were eluted with 50 µl of sample buffer by boiling, and 20 µl of immunoprecipitates and 4% of whole cell extracts, used for the loading control, were resolved on 4–12% NuPAGE (Invitrogen) and immunoblotted with indicated antibodies as described [40]. For SUMOylation assay, 293T cells transfected with pcDNA3.1 for IRF7 or IRF3, PIAS1, VP35 along with V5-SUMO3 for 30 h. Extract preparations, immunoprecipitation and immunoblotting were performed according to the described method [72].

### Luciferase reporter analysis

293T or A549 cells were transfected with the indicated amounts of pGL4 vector with the IFNβ promoter and pRL-TK reporters along with other expression vectors using the FuGENE 6 Transfection Reagent (Roche) for 24 h, and were infected with NDV for 24 h [40]. Lysates were analyzed for luciferase activity using the dual-luciferase assays kit (Promega). IFNβ reporter activity was normalized by Renilla luciferase activity.

## Supporting Information

Figure S1 VP35 from Zaire EBOV (hVP35) and mouse-adapted EBOV (mVP35) both inhibit type I IFN expression in murine DCs. (A) VP35 from human and mouse EBOVs were tagged to EGFP and cloned in pcDNA3.1 and transfected into 293T cells (3×10^5^ cells). Whole cell extracts harvested 24 h after transfection were tested by immunoblot using anti-GFP antibody. (B) BMDCs were transduced with pMSCV with hVP35-EGFP or free EGFP (Ctrl) on day 2; cells were stimulated with NDV on day 8 for 5 h. IFNα proteins and transcripts were measured by ELISA and qRT-PCR, respectively. Values represent the average of three assays+/−S.D. (C) Above DCs were stimulated with NDV or CpG DNA and Ifn{lower case betaa} or Ifnα transcripts were measured as above. (D) DCs transduced with hVP35-HA, mVP35-HA or free EGFP (Ctrl) were infected with NDV for 7 h and chromatin was precipitated with anti-IRF7 antibody (solid bar) or normal rabbit IgG (open bar). Precipitated DNA was amplified for the Ifna4 and Ifnb promoters by q-PCR. ChIP signals are expressed as the percentage of input DNA (1%).
(2.31 MB TIF)

Figure S2 Interaction of VP35 with IRF7. (A) VP35 interacts with IRF7 before and after NDV infection. 293T cells (1×10^6^) were transfected with pcDNA3.1 vector for Flag-IRF7 (2 µg) and VP35-HA (2 µg) for 24 h and infected with or without NDV for 24 h. Extracts were precipitated with anti-Flag antibody and blotted with anti-HA antibody. Whole cell extracts (WCE) were blotted with indicated antibodies to verify expression of transfected proteins. (B) VP35 binds to two separate domains of IRF7. Schematic diagram of IRF7 deletions. Results of domain analysis are summarized on right. (C) 293T cells were cotransfected with pcDNA3.1 vector for VP35-HA (2 µg) and Flag-IRF7 deletion constructs (2 µg) for 24 h. The extracts were immunoblotted with indicated antibodies. The two regions through which VP35 interacts with IRF7 are predicted to juxtapose in crystallography [47].
(1.90 MB TIF)

Figure S3 PIAS1 mediates SUMO1 conjugation to IRF7. 293T cells were transfected with T7-tagged SUMO1 (0.5 µg), PIAS1-HA (2 µg) along with Flag-IRF7 or Flag- IRF7 6D (1 µg) for 30 h. Extracts were precipitated with anti-Flag antibody and blotted with antibody to T7.
(0.64 MB TIF)

Figure S4 VP35 triggers IRF7 SUMOylation. (A) 293T cells (1×10^6^) were transfected with V5-tagged SUMO3 (0.5 µg), Flag-IRF7 (1 µg) and VP35-HA (0.5 and 1 µg) for 30 h. Extracts were precipitated with antibody to Flag and blotted with antibody to V5. (B) Cells were transfected with V5-tagged SUMO3 (0.5 µg), VP35-HA (2 µg) along with Flag-IRF7, Flag-IRF7 K406R or Flag-IRF7 K43R (1 µg) and tested as above.
(1.66 MB TIF)

Figure S5 VP35 enhances IRF3 SUMOylation. (A) Interaction of VP35 with IRF3. Cells (1×10^6^) were transfected with HA-tagged VP35 (2 µg) Flag- IRF3 (2 µg each) and extracts were precipitated with anti-Flag antibody, blotted with anti-HA antibody. (B) 293T cells were transfected with V5-tagged SUMO3 (0.5 µg), VP35-HA (2 µg) along with Flag-IRF3 or Flag-IRF3 5D (1 µg) for 30 h. Extracts were precipitated with antibody to Flag and blotted with antibody to V5. (C) 293T cells (1×10^6^) were transfected with increasing doses of VP35-HA (0.5 µg, 1 µg and 2 µg) along with Flag-IRF3 5D (1 µg) for 30 h. Extracts were precipitated with antibody to Flag and blotted with antibody to V5. (D) Cells were transfected with VP35-HA (0.5 µg) or IRF3 5D alone (0.1 µg) or together, along with IFNβ reporter plus pRL-TK for 24 h and then NDV infection. Post infection 24 h, the cell lysates were harvested for dual Luciferase activity. (E) The Luciferase assay was performed as in (C). The VP35 inhibition of IRF3 and IRF3 5D activity was calculated relative to the control activity without VP35 expression.
(1.67 MB TIF)

Table S1 The potential SUMO conjugation sites in the mouse IRF7 predicted by the SUMOsp software by Xue Y., et al (http://sumosp.biocuckoo.org/) [56]. Previously, only K406 was shown to be SUMOylated [40]. However, our data in Figure 7 indicate that VP35 promotes SUMO conjugation at this and additional sites, supported by this prediction.
(0.03 MB DOC)

## References

[1] Feldmann H, Jones S, Klenk HD, Schnittler HJ (2003). Ebola virus: from discovery to vaccine.. Nat Rev Immunol.

[2] Bray M, Geisbert TW (2005). Ebola virus: the role of macrophages and dendritic cells in the pathogenesis of Ebola hemorrhagic fever.. Int J Biochem Cell Biol.

[3] Bosio CM, Aman MJ, Grogan C, Hogan R, Ruthel G (2003). Ebola and Marburg viruses replicate in monocyte-derived dendritic cells without inducing the production of cytokines and full maturation.. J Infect Dis.

[4] Mahanty S, Hutchinson K, Agarwal S, McRae M, Rollin PE (2003). Cutting edge: impairment of dendritic cells and adaptive immunity by Ebola and Lassa viruses.. J Immunol.

[5] Geisbert TW, Hensley LE, Larsen T, Young HA, Reed DS (2003). Pathogenesis of Ebola hemorrhagic fever in cynomolgus macaques: evidence that dendritic cells are early and sustained targets of infection.. Am J Pathol.

[6] Hutchinson KL, Rollin PE (2007). Cytokine and chemokine expression in humans infected with Sudan Ebola virus.. J Infect Dis.

[7] Mohamadzadeh M, Chen L, Schmaljohn AL (2007). How Ebola and Marburg viruses battle the immune system.. Nat Rev Immunol.

[8] Feldmann H, Jones SM, Daddario-DiCaprio KM, Geisbert JB, Stroher U (2007). Effective post-exposure treatment of Ebola infection.. PLoS Pathog.

[9] Bradfute S, Kelley L, Bavari S (2008). Functional CD8+T cell response in lethal Ebola virus infection.. J Immunol.

[10] Bray M (2001). The role of the Type I interferon response in the resistance of mice to filovirus infection.. J Gen Virol.

[11] Mahanty S, Gupta M, Paragas J, Bray M, Ahmed R (2003). Protection from lethal infection is determined by innate immune responses in a mouse model of Ebola virus infection.. Virology.

[12] Jahrling PB, Geisbert TW, Geisbert JB, Swearengen JR, Bray M (1999). Evaluation of immune globulin and recombinant interferon-alpha2b for treatment of experimental Ebola virus infections.. J Infect Dis.

[13] Basler CF, Wang X, Muhlberger E, Volchkov V, Paragas J (2000). The Ebola virus VP35 protein functions as a type I IFN antagonist.. Proc Natl Acad Sci U S A.

[14] Basler CF, Mikulasova A, Martinez-Sobrido L, Paragas J, Muhlberger E (2003). The Ebola virus VP35 protein inhibits activation of interferon regulatory factor 3.. J Virol.

[15] Reid SP, Valmas C, Martinez O, Sanchez FM, Basler CF (2007). Ebola virus VP24 proteins inhibit the interaction of NPI-1 subfamily karyopherin alpha proteins with activated STAT1.. J Virol.

[16] Reid SP, Leung LW, Hartman AL, Martinez O, Shaw ML (2006). Ebola virus VP24 binds karyopherin alpha1 and blocks STAT1 nuclear accumulation.. J Virol.

[17] Cardenas WB, Loo YM, Gale M, Hartman AL, Kimberlin CR (2006). Ebola virus VP35 protein binds double-stranded RNA and inhibits alpha/beta interferon production induced by RIG-I signaling.. J Virol.

[18] Feng Z, Cerveny M, Yan Z, He B (2007). The VP35 protein of Ebola virus inhibits the antiviral effect mediated by double-stranded RNA-dependent protein kinase PKR.. J Virol.

[19] Hartman AL, Bird BH, Towner JS, Antoniadou ZA, Zaki SR (2008). Inhibition of IRF-3 activation by VP35 is critical for the high level of virulence of ebola virus.. J Virol.

[20] Hartman AL, Ling L, Nichol ST, Hibberd ML (2008). Whole-genome expression profiling reveals that inhibition of host innate immune response pathways by Ebola virus can be reversed by a single amino acid change in the VP35 protein.. J Virol.

[21] Prins KC, Cardenas WB, Basler CF (2009). Ebola virus protein VP35 impairs the function of interferon regulatory factor-activating kinases IKKepsilon and TBK-1.. J Virol.

[22] Honda K, Taniguchi T (2006). IRFs: master regulators of signalling by Toll-like receptors and cytosolic pattern-recognition receptors.. Nat Rev Immunol.

[23] Honda K, Yanai H, Negishi H, Asagiri M, Sato M (2005). IRF-7 is the master regulator of type-I interferon-dependent immune responses.. Nature.

[24] Takeuchi O, Akira S (2007). Recognition of viruses by innate immunity.. Immunol Rev.

[25] Solis M, Goubau D, Romieu-Mourez R, Genin P, Civas A (2006). Distinct functions of IRF-3 and IRF-7 in IFN-alpha gene regulation and control of anti-tumor activity in primary macrophages.. Biochem Pharmacol.

[26] Tamura T, Yanai H, Savitsky D, Taniguchi T (2008). The IRF family transcription factors in immunity and oncogenesis.. Annu Rev Immunol.

[27] Asselin-Paturel C, Trinchieri G (2005). Production of type I interferons: plasmacytoid dendritic cells and beyond.. J Exp Med.

[28] Dai J, Megjugorac NJ, Amrute SB, Fitzgerald-Bocarsly P (2004). Regulation of IFN regulatory factor-7 and IFN-alpha production by enveloped virus and lipopolysaccharide in human plasmacytoid dendritic cells.. J Immunol.

[29] Gabriele L, Ozato K (2007). The role of IRF family in dendritic cell development and function.. Cytokine Growth Factor Review in press.

[30] Shortman K, Naik SH (2007). Steady-state and inflammatory dendritic-cell development.. Nat Rev Immunol.

[31] Steinman RM, Hemmi H (2006). Dendritic cells: translating innate to adaptive immunity.. Curr Top Microbiol Immunol.

[32] Kawai T, Akira S (2007). TLR signaling.. Semin Immunol.

[33] Liu YJ (2005). IPC: professional type 1 interferon-producing cells and plasmacytoid dendritic cell precursors.. Annu Rev Immunol.

[34] Honda K, Ohba Y, Yanai H, Negishi H, Mizutani T (2005). Spatiotemporal regulation of MyD88-IRF-7 signalling for robust type-I interferon induction.. Nature.

[35] Kawai T, Sato S, Ishii KJ, Coban C, Hemmi H (2004). Interferon-alpha induction through Toll-like receptors involves a direct interaction of IRF7 with MyD88 and TRAF6.. Nat Immunol.

[36] Tailor P, Tamura T, Kong HJ, Kubota T, Kubota M (2007). The feedback phase of type I interferon induction in dendritic cells requires interferon regulatory factor 8.. Immunity.

[37] Hay RT (2005). SUMO: a history of modification.. Mol Cell.

[38] Meulmeester E, Melchior F (2008). Cell biology: SUMO.. Nature.

[39] Geiss-Friedlander R, Melchior F (2007). Concepts in sumoylation: a decade on.. Nat Rev Mol Cell Biol.

[40] Kubota T, Matsuoka M, Chang TH, Tailor P, Sasaki T (2008). Virus infection triggers SUMOylation of IRF3 and IRF7, leading to the negative regulation of type I interferon gene expression.. J Biol Chem.

[41] Tamura T, Tailor P, Yamaoka K, Kong HJ, Tsujimura H (2005). IFN regulatory factor-4 and -8 govern dendritic cell subset development and their functional diversity.. J Immunol.

[42] Saccani S, Natoli G (2002). Dynamic changes in histone H3 Lys 9 methylation occurring at tightly regulated inducible inflammatory genes.. Genes Dev.

[43] Ghosh S, Karin M (2002). Missing pieces in the NF-kappaB puzzle.. Cell.

[44] Liu B, Liao J, Rao X, Kushner SA, Chung CD (1998). Inhibition of Stat1-mediated gene activation by PIAS1.. Proc Natl Acad Sci U S A.

[45] Liu B, Mink S, Wong KA, Stein N, Getman C (2004). PIAS1 selectively inhibits interferon-inducible genes and is important in innate immunity.. Nat Immunol.

[46] Hammer E, Heilbronn R, Weger S (2007). The E3 ligase Topors induces the accumulation of polysumoylated forms of DNA topoisomerase I in vitro and in vivo.. FEBS Lett.

[47] Qin BY, Liu C, Lam SS, Srinath H, Delston R (2003). Crystal structure of IRF-3 reveals mechanism of autoinhibition and virus-induced phosphoactivation.. Nat Struct Biol.

[48] Gill G (2005). Something about SUMO inhibits transcription.. Curr Opin Genet Dev.

[49] Lin R, Heylbroeck C, Pitha PM, Hiscott J (1998). Virus-dependent phosphorylation of the IRF-3 transcription factor regulates nuclear translocation, transactivation potential, and proteasome-mediated degradation.. Mol Cell Biol.

[50] Tatham MH, Jaffray E, Vaughan OA, Desterro JM, Botting CH (2001). Polymeric chains of SUMO-2 and SUMO-3 are conjugated to protein substrates by SAE1/SAE2 and Ubc9.. J Biol Chem.

[51] Yang M, Hsu CT, Ting CY, Liu LF, Hwang J (2006). Assembly of a polymeric chain of SUMO1 on human topoisomerase I in vitro.. J Biol Chem.

[52] Dobreva G, Dambacher J, Grosschedl R (2003). SUMO modification of a novel MAR-binding protein, SATB2, modulates immunoglobulin mu gene expression.. Genes Dev.

[53] Sapetschnig A, Rischitor G, Braun H, Doll A, Schergaut M (2002). Transcription factor Sp3 is silenced through SUMO modification by PIAS1.. Embo J.

[54] Rogers RS, Horvath CM, Matunis MJ (2003). SUMO modification of STAT1 and its role in PIAS-mediated inhibition of gene activation.. J Biol Chem.

[55] Lee JM, Kang HJ, Lee HR, Choi CY, Jang WJ (2003). PIAS1 enhances SUMO-1 modification and the transactivation activity of the major immediate-early IE2 protein of human cytomegalovirus.. FEBS Lett.

[56] Xue Y, Zhou F, Fu C, Xu Y, Yao X (2006). SUMOsp: a web server for sumoylation site prediction.. Nucleic Acids Res.

[57] Haller O, Weber F (2007). Pathogenic viruses: smart manipulators of the interferon system.. Curr Top Microbiol Immunol.

[58] Ozato K, Tailor P, Kubota T (2007). The interferon regulatory factor family in host defense: mechanism of action.. J Biol Chem.

[59] Higgs R, Ni Gabhann J, Ben Larbi N, Breen EP, Fitzgerald KA (2008). The E3 ubiquitin ligase Ro52 negatively regulates IFN-beta production post-pathogen recognition by polyubiquitin-mediated degradation of IRF3.. J Immunol.

[60] Boutell C, Orr A, Everett RD (2003). PML residue lysine 160 is required for the degradation of PML induced by herpes simplex virus type 1 regulatory protein ICP0.. J Virol.

[61] Muller S, Dejean A (1999). Viral immediate-early proteins abrogate the modification by SUMO-1 of PML and Sp100 proteins, correlating with nuclear body disruption.. J Virol.

[62] Boggio R, Passafaro A, Chiocca S (2007). Targeting SUMO E1 to ubiquitin ligases: a viral strategy to counteract sumoylation.. J Biol Chem.

[63] Chiu MW, Shih HM, Yang TH, Yang YL (2007). The type 2 dengue virus envelope protein interacts with small ubiquitin-like modifier-1 (SUMO-1) conjugating enzyme 9 (Ubc9).. J Biomed Sci.

[64] Wormald S, Hilton DJ (2004). Inhibitors of cytokine signal transduction.. J Biol Chem.

[65] Sharrocks AD (2006). PIAS proteins and transcriptional regulation–more than just SUMO E3 ligases?. Genes Dev.

[66] Nakagawa K, Yokosawa H (2002). PIAS3 induces SUMO-1 modification and transcriptional repression of IRF-1.. FEBS Lett.

[67] Choi CY, Kim YH, Kwon HJ, Kim Y (1999). The homeodomain protein NK-3 recruits Groucho and a histone deacetylase complex to repress transcription.. J Biol Chem.

[68] Ross S, Best JL, Zon LI, Gill G (2002). SUMO-1 modification represses Sp3 transcriptional activation and modulates its subnuclear localization.. Mol Cell.

[69] Pascual G, Fong AL, Ogawa S, Gamliel A, Li AC (2005). A SUMOylation-dependent pathway mediates transrepression of inflammatory response genes by PPAR-gamma.. Nature.

[70] Mochizuki K, Nishiyama A, Jang MK, Dey A, Ghosh A (2008). The bromodomain protein Brd4 stimulates G1 gene transcription and promotes progression to S phase.. J Biol Chem.

[71] Tsujimura H, Tamura T, Kong HJ, Nishiyama A, Ishii KJ (2004). Toll-like receptor 9 signaling activates NF-kappaB through IFN regulatory factor-8/IFN consensus sequence binding protein in dendritic cells.. J Immunol.

[72] Jaffray EG, Hay RT (2006). Detection of modification by ubiquitin-like proteins.. Methods.

