# Supplementary figures and images for: Ebola Zaire Virus Blocks Type I Interferon Production by Exploiting the Host SUMO Modification Machinery
Source: PLoS Pathog. 2009 Jun 26;5(6):e1000493. doi: 10.1371/journal.ppat.1000493 (PMC2696038; doi:10.1371/journal.ppat.1000493)

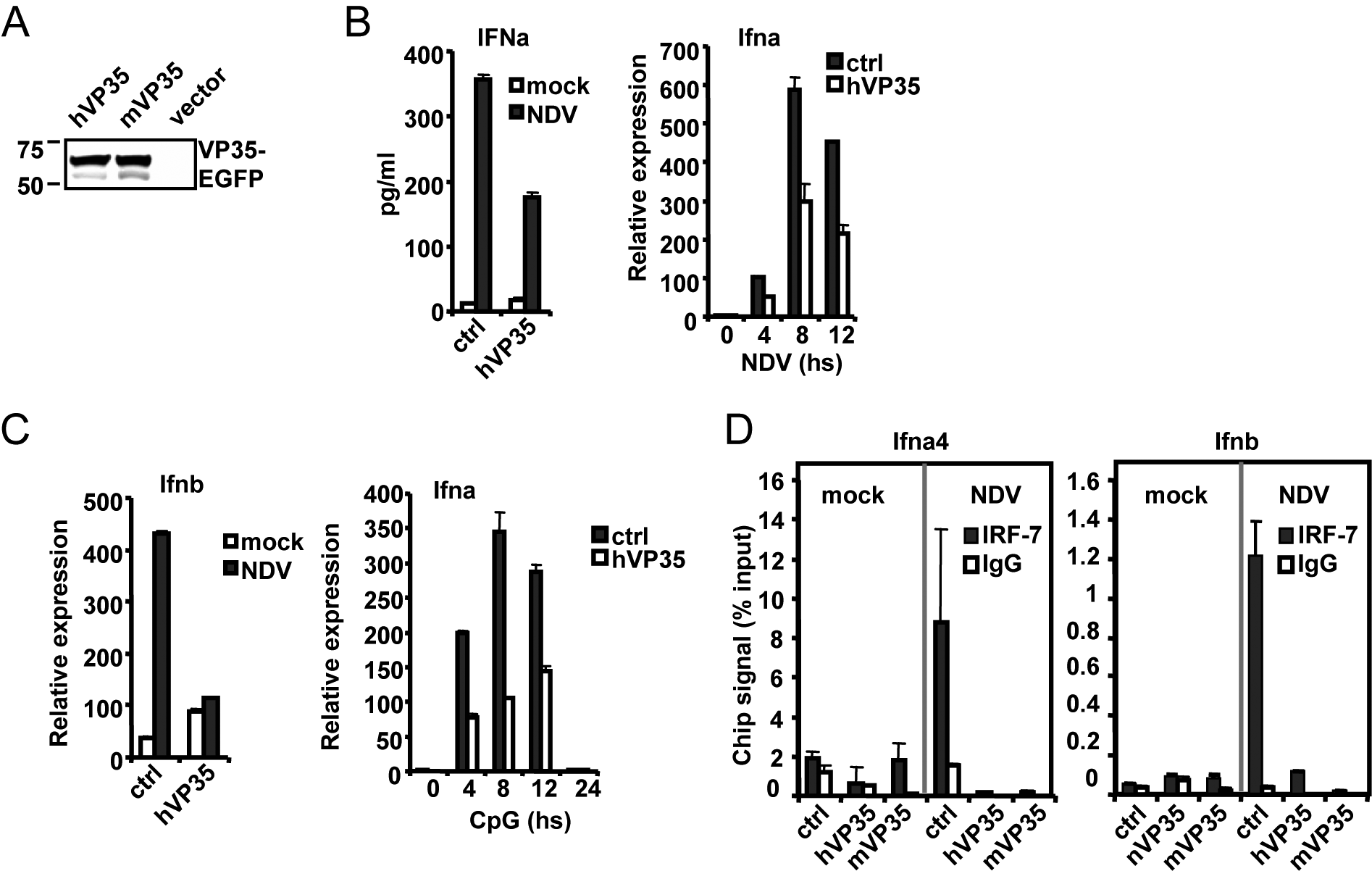

Supplement: Figure S1 — VP35 from Zaire EBOV (hVP35) and mouse-adapted EBOV (mVP35) both inhibit type I IFN expression in murine DCs. (A) VP35 from human and mouse EBOVs were tagged to EGFP and cloned in pcDNA3.1 and transfected into 293T cells (3×10^5^ cells). Whole cell extracts harvested 24 h after transfection were tested by immunoblot using anti-GFP antibody. (B) BMDCs were transduced with pMSCV with hVP35-EGFP or free EGFP (Ctrl) on day 2; cells were stimulated with NDV on day 8 for 5 h. IFNα proteins and transcripts were measured by ELISA and qRT-PCR, respectively. Values represent the average of three assays+/−S.D. (C) Above DCs were stimulated with NDV or CpG DNA and Ifn{lower case betaa} or Ifnα transcripts were measured as above. (D) DCs transduced with hVP35-HA, mVP35-HA or free EGFP (Ctrl) were infected with NDV for 7 h and chromatin was precipitated with anti-IRF7 antibody (solid bar) or normal rabbit IgG (open bar). Precipitated DNA was amplified for the Ifna4 and Ifnb promoters by q-PCR. ChIP signals are expressed as the percentage of input DNA (1%). (2.31 MB TIF) [file ppat.1000493.s001.tif]

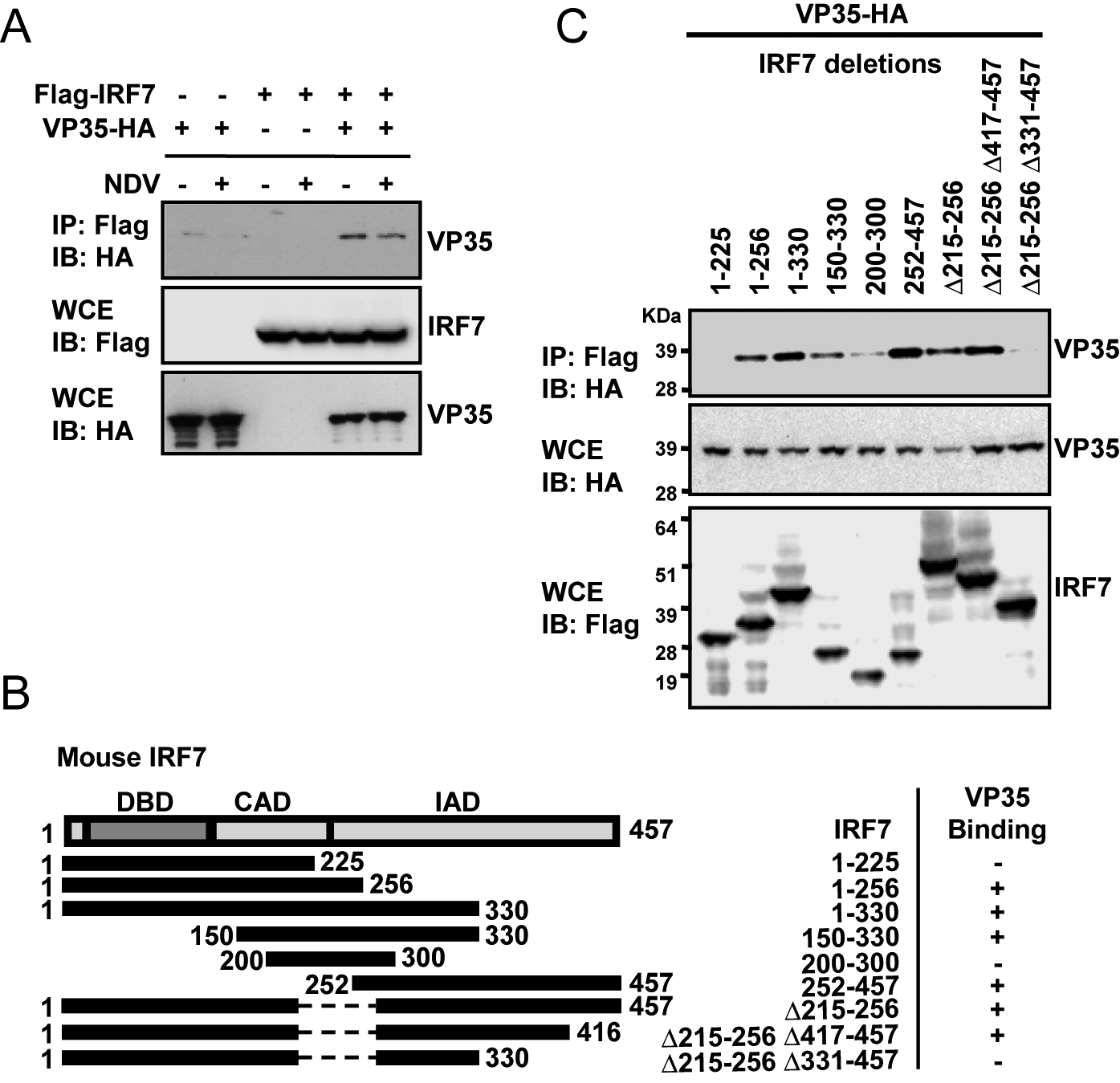

Supplement: Figure S2 — Interaction of VP35 with IRF7. (A) VP35 interacts with IRF7 before and after NDV infection. 293T cells (1×10^6^) were transfected with pcDNA3.1 vector for Flag-IRF7 (2 µg) and VP35-HA (2 µg) for 24 h and infected with or without NDV for 24 h. Extracts were precipitated with anti-Flag antibody and blotted with anti-HA antibody. Whole cell extracts (WCE) were blotted with indicated antibodies to verify expression of transfected proteins. (B) VP35 binds to two separate domains of IRF7. Schematic diagram of IRF7 deletions. Results of domain analysis are summarized on right. (C) 293T cells were cotransfected with pcDNA3.1 vector for VP35-HA (2 µg) and Flag-IRF7 deletion constructs (2 µg) for 24 h. The extracts were immunoblotted with indicated antibodies. The two regions through which VP35 interacts with IRF7 are predicted to juxtapose in crystallography [47]. (1.90 MB TIF) [file ppat.1000493.s002.tif]

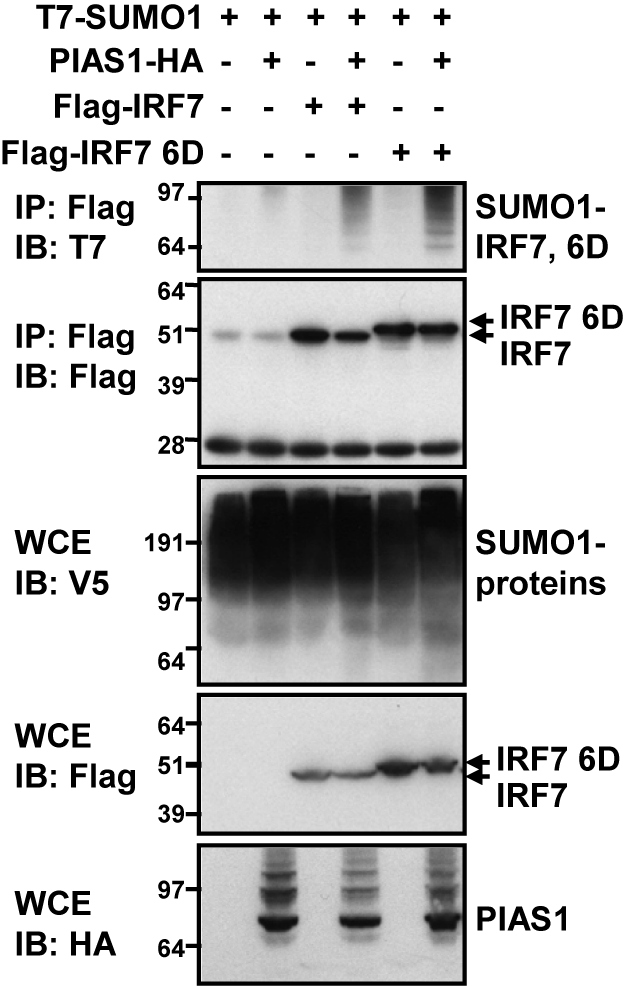

Supplement: Figure S3 — PIAS1 mediates SUMO1 conjugation to IRF7. 293T cells were transfected with T7-tagged SUMO1 (0.5 µg), PIAS1-HA (2 µg) along with Flag-IRF7 or Flag- IRF7 6D (1 µg) for 30 h. Extracts were precipitated with anti-Flag antibody and blotted with antibody to T7. (0.64 MB TIF) [file ppat.1000493.s003.tif]

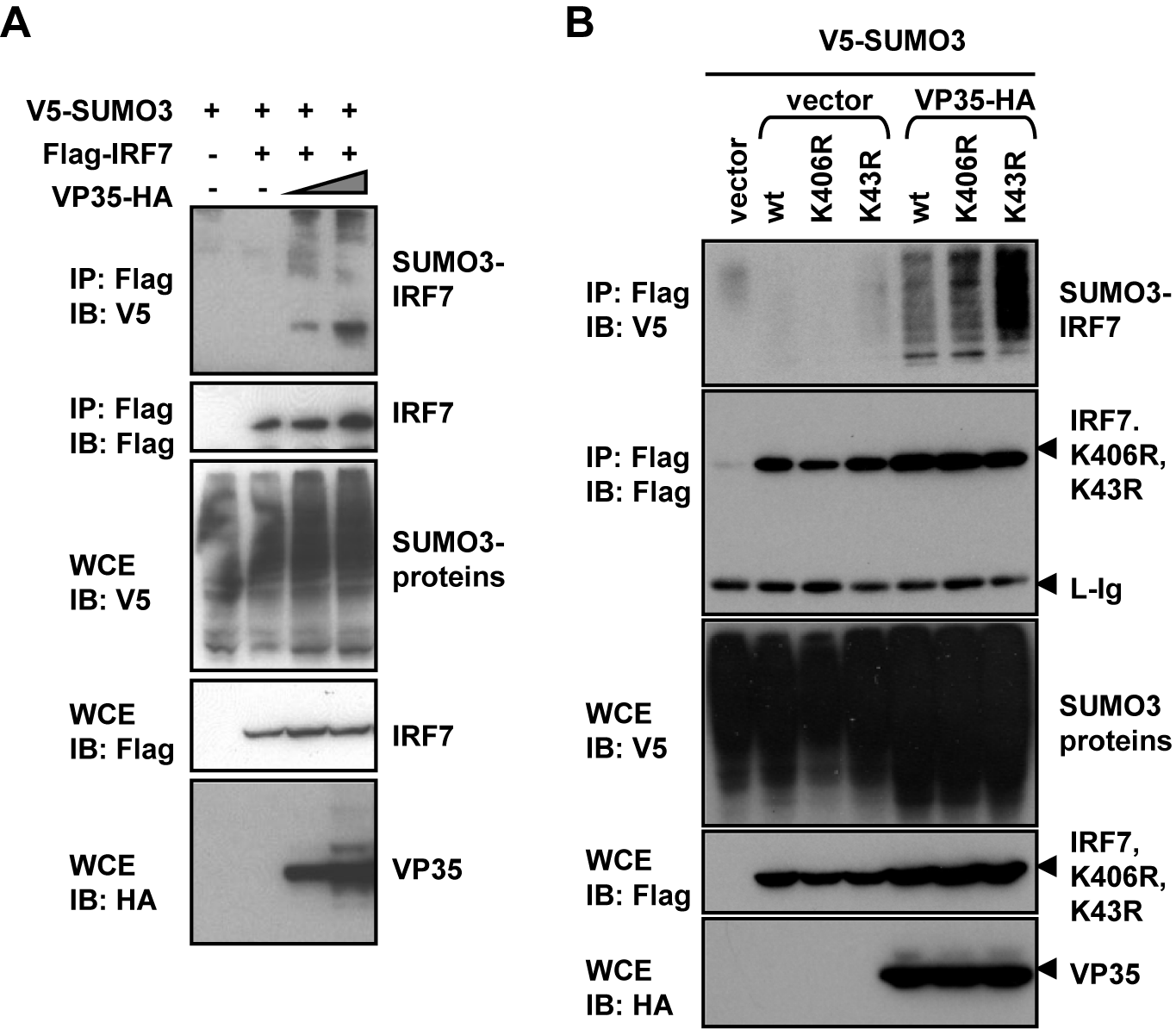

Supplement: Figure S4 — VP35 triggers IRF7 SUMOylation. (A) 293T cells (1×10^6^) were transfected with V5-tagged SUMO3 (0.5 µg), Flag-IRF7 (1 µg) and VP35-HA (0.5 and 1 µg) for 30 h. Extracts were precipitated with antibody to Flag and blotted with antibody to V5. (B) Cells were transfected with V5-tagged SUMO3 (0.5 µg), VP35-HA (2 µg) along with Flag-IRF7, Flag-IRF7 K406R or Flag-IRF7 K43R (1 µg) and tested as above. (1.66 MB TIF) [file ppat.1000493.s004.tif]

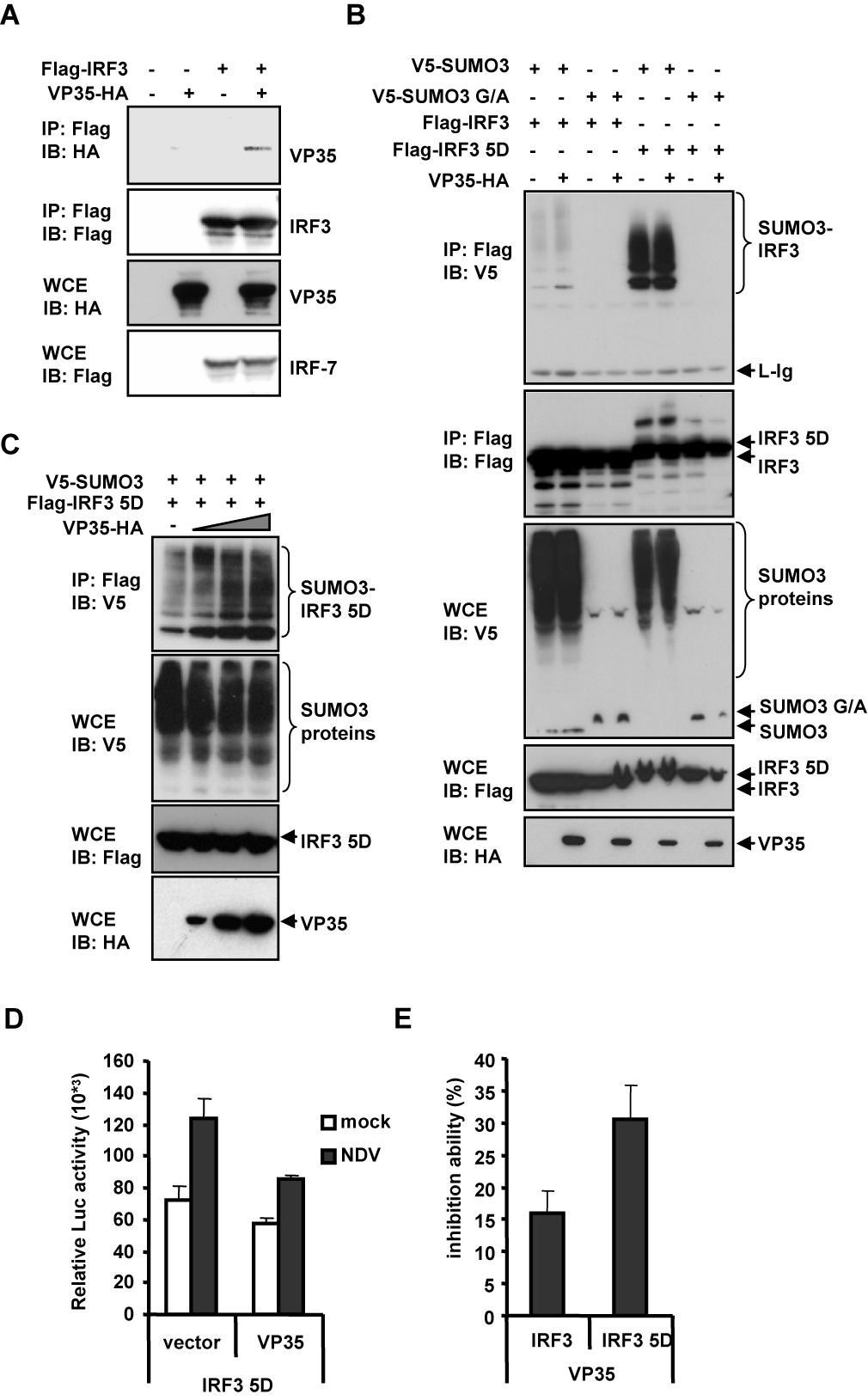

Supplement: Figure S5 — VP35 enhances IRF3 SUMOylation. (A) Interaction of VP35 with IRF3. Cells (1×10^6^) were transfected with HA-tagged VP35 (2 µg) Flag- IRF3 (2 µg each) and extracts were precipitated with anti-Flag antibody, blotted with anti-HA antibody. (B) 293T cells were transfected with V5-tagged SUMO3 (0.5 µg), VP35-HA (2 µg) along with Flag-IRF3 or Flag-IRF3 5D (1 µg) for 30 h. Extracts were precipitated with antibody to Flag and blotted with antibody to V5. (C) 293T cells (1×10^6^) were transfected with increasing doses of VP35-HA (0.5 µg, 1 µg and 2 µg) along with Flag-IRF3 5D (1 µg) for 30 h. Extracts were precipitated with antibody to Flag and blotted with antibody to V5. (D) Cells were transfected with VP35-HA (0.5 µg) or IRF3 5D alone (0.1 µg) or together, along with IFNβ reporter plus pRL-TK for 24 h and then NDV infection. Post infection 24 h, the cell lysates were harvested for dual Luciferase activity. (E) The Luciferase assay was performed as in (C). The VP35 inhibition of IRF3 and IRF3 5D activity was calculated relative to the control activity without VP35 expression. (1.67 MB TIF) [file ppat.1000493.s005.tif]
