# Supplementary material for: Ebola Zaire Virus Blocks Type I Interferon Production by Exploiting the Host SUMO Modification Machinery
Source: PLoS Pathog. 2009 Jun 26;5(6):e1000493. doi: 10.1371/journal.ppat.1000493 (PMC2696038; doi:10.1371/journal.ppat.1000493)
Supplement: Table S1 — The potential SUMO conjugation sites in the mouse IRF7 predicted by the SUMOsp software by Xue Y., et al (http://sumosp.biocuckoo.org/) [56]. Previously, only K406 was shown to be SUMOylated [40]. However, our data in Figure 7 indicate that VP35 promotes SUMO conjugation at this and additional sites, supported by this prediction. (0.03 MB DOC) [file ppat.1000493.s006.doc]

**Table S1.** SUMO conjugation sites predicted for the mouse IRF7*

| **Lysine position** | **Motif** |
| --- | --- |
| 43 | VPWKHFG |
| 59 | QIFKAWA |
| 92 | RGWKTNF |
| 120 | DPHKVYE |
| 250 | IMYKGRT |
| 295 | PDQKQLH |
| 327 | RMGKCKV |
| 329 | GKCKVYW |
| 398 | GRPKEKT |
| 400 | PKEKTLI |
| 406 | ILVKLEP |
| 413 | WVCKAYL |

* Based on the model by Ren J. and Xue Y., et al (56)
